# Isolation and characterization of cross-neutralizing coronavirus antibodies from COVID-19+ subjects

**DOI:** 10.1016/j.celrep.2021.109353

**Published:** 2021-06-22

**Authors:** Madeleine F. Jennewein, Anna J. MacCamy, Nicholas R. Akins, Junli Feng, Leah J. Homad, Nicholas K. Hurlburt, Emilie Seydoux, Yu-Hsin Wan, Andrew B. Stuart, Venkata Viswanadh Edara, Katharine Floyd, Abigail Vanderheiden, John R. Mascola, Nicole Doria-Rose, Lingshu Wang, Eun Sung Yang, Helen Y. Chu, Jonathan L. Torres, Gabriel Ozorowski, Andrew B. Ward, Rachael E. Whaley, Kristen W. Cohen, Marie Pancera, M. Juliana McElrath, Janet A. Englund, Andrés Finzi, Mehul S. Suthar, Andrew T. McGuire, Leonidas Stamatatos

**Affiliations:** 1 Fred Hutchinson Cancer Research Center, Vaccines and Infectious Disease Division, Seattle, WA 98109, USA; 2 Center for Childhood Infections and Vaccines of Children’s Healthcare of Atlanta, Department of Pediatrics, Emory University School of Medicine, Emory Vaccine Center, Yerkes National Primate Research Center, Atlanta, GA 30322, USA; 3 Department of Integrative Structural and Computational Biology, The Scripps Research Institute, La Jolla, CA 92037, USA; 4 Vaccine Research Center, NIAID, NIH, Bethesda, MD 20892, USA; 5 University of Washington, Department of Medicine, Seattle, WA 98109, USA; 6 University of Washington, Department of Global Health, Seattle, WA 98109, USA; 7 Department of Pediatrics, University of Washington and Seattle Children’s Research Institute, Seattle, WA 98109, USA; 8 Université de Montréal, Montreal, QC, Canada; 9 Department of Laboratory Medicine and Pathology, University of Washington, Seattle, WA 98195, USA

**Keywords:** SARS-CoV-2, SARS-CoV-1, S2 subunit, RBD, NTD, neutralization, monoclonal antibodies, B.1.351, CV3-25

## Abstract

SARS-CoV-2 is one of three coronaviruses that have crossed the animal-to-human barrier and caused widespread disease in the past two decades. The development of a universal human coronavirus vaccine could prevent future pandemics. We characterize 198 antibodies isolated from four COVID-19+ subjects and identify 14 SARS-CoV-2 neutralizing antibodies. One targets the N-terminal domain (NTD), one recognizes an epitope in S2, and 11 bind the receptor-binding domain (RBD). Three anti-RBD neutralizing antibodies cross-neutralize SARS-CoV-1 by effectively blocking binding of both the SARS-CoV-1 and SARS-CoV-2 RBDs to the ACE2 receptor. Using the K18-hACE transgenic mouse model, we demonstrate that the neutralization potency and antibody epitope specificity regulates the *in vivo* protective potential of anti-SARS-CoV-2 antibodies. All four cross-neutralizing antibodies neutralize the B.1.351 mutant strain. Thus, our study reveals that epitopes in S2 can serve as blueprints for the design of immunogens capable of eliciting cross-neutralizing coronavirus antibodies.

## Highlights

• Fourteen anti-SARS-CoV-2 neutralizing mAbs isolated from four patients
• Three anti-RBD and one anti-S2 mAb neutralized SARS-CoV-1 and the B.1.351 variant
• Mouse studies show potential protective effect of anti-NTD mAbs

## Graphical abstract

**Figure:**
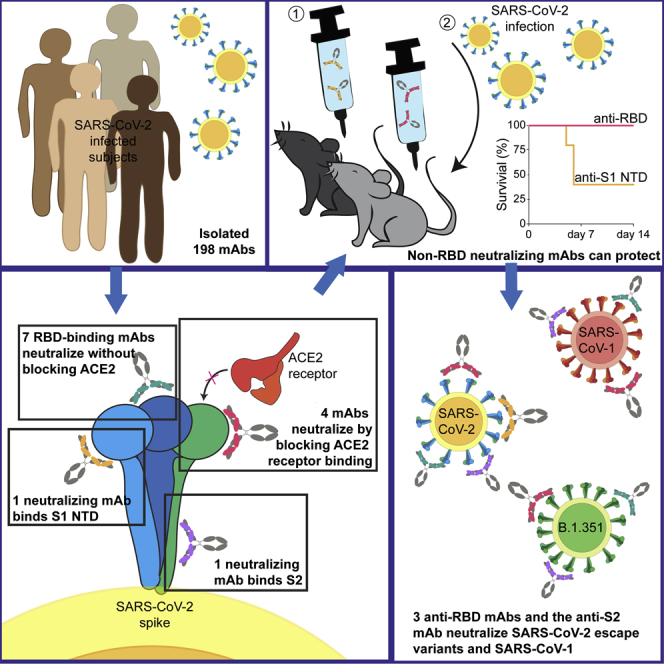

## Teaser

Jennewein et al. isolated 14 anti-SARS-CoV-2 neutralizing antibodies—one anti-S2, one anti-NTD, and 11 anti-RBD—from four patients. Three anti-RBD and the anti-S2 nAbs cross-neutralized SARS-CoV-1 and B.1.351. The anti-NTD mAbs conferred partial protection in mice. Evolved anti-S2 Abs may provide templates for pan-coronavirus vaccines.

## Introduction

In the past two decades, there have been zoonotic transmissions of three highly pathogenic coronaviruses—SARS-CoV-1, MERS-CoV, and SARS-CoV-2—which have caused widespread human disease. The most recent one, SARS-CoV-2, has been rapidly spreading globally since late 2019/early 2020, infecting over 160 million people and killing almost 3.4 million people by May 2021 (Dong et al., 2020; Patel et al., 2020). Studies conducted in mice, hamsters, and non-human primates strongly suggest that neutralizing antibodies (nAbs) isolated from infected patients can protect infection and, in the case of established infection, can reduce viremia and mitigate the development of clinical symptoms (Baum et al., 2020b; Cao et al., 2020a; Mercado et al., 2020; Rogers et al., 2020a; Schäfer et al., 2021; Shi et al., 2020; Tortorici et al., 2020; Wu et al., 2020; Yu et al., 2020). Cocktails of neutralizing monoclonal Abs (mAbs) have been approved by the FDA for the treatment of infection (Baum et al., 2020a; Weinreich et al., 2020). Thus, nAbs are believed to be an important component of the protective immune responses elicited by effective vaccines. Indeed, both the mRNA-based Pfizer and Moderna vaccines elicit potent serum neutralizing Ab responses against SARS-CoV-2 (Jackson et al., 2020; Walsh et al., 2020).

mAbs with neutralizing activities have been isolated from infected patients, and their characterization led to the identification of vulnerable sites on the viral spike protein (S) (Cao et al., 2020a; Ju et al., 2020; Kreer et al., 2020; Liu et al., 2020; Nielsen et al., 2020; Robbiani et al., 2020; Seydoux et al., 2020; Wan et al., 2020a; Zost et al., 2020).

Many known SARS-CoV-2 nAbs bind the receptor-binding domain (RBD) and block its interaction with its cellular receptor, angiotensin-converting enzyme 2 (ACE2), thus preventing viral attachment and cell fusion (Hoffmann et al., 2020; Yan et al., 2020). However, some RBD-binding mAbs prevent infection without interfering with the RBD-ACE2 interaction (Pinto et al., 2020; Tai et al., 2020; Wang et al., 2020a). Other mAbs neutralize without binding to the RBD (Brouwer et al., 2020; Cerutti et al., 2021; Chi et al., 2020; Liu et al., 2020; McCallum et al., 2021; Song et al., 2020; Wang et al., 2020a, 2020b), and their mechanisms of action are not fully understood (Gavor et al., 2020).

Plasma from SARS-CoV-1- and SARS-CoV-2-infected people may contain cross-reactive binding Abs (Ju et al., 2020; Lv et al., 2020), and a small number of mAbs that can neutralize both viruses have been isolated from SARS-CoV-2- (Brouwer et al., 2020; Rogers et al., 2020a; Wec et al., 2020) or SARS-CoV-1-infected subjects (Tortorici et al., 2020). Overall, it appears that most cross-reactive Abs do not cross-neutralize and that cross-nAbs are infrequently generated during SARS-CoV-2 or SARS-CoV-1 infections. Abs capable of neutralizing SARS-CoV-1, SARS-CoV-2, and endemic human coronaviruses, such as the betacoronaviruses OC43 and HKU1 or the alphacoronaviruses 229E and NL63, have not yet been identified.

Here, we report on the isolation and full characterization of 198 S-specific mAbs from four SARS-CoV-2-infected individuals. Although a number of these mAbs recognized both SARS-CoV-2 and SARS-CoV-1, we observed minimal cross-reactivity with MERS-CoV, betacoronaviruses (OC43 and HKU1), or alphacoronaviruses (NL63 and 229E). A significant fraction of cross-reactive Abs bound the SARS-CoV-2 S2 domain of the S protein. Fourteen mAbs neutralized SARS-CoV-2. One neutralizing mAb bound the NTD, one bound the S2 subunit, one bound an unidentified site on S, and the remaining 11 bound the RBD. Some competed with the RBD-ACE-2 interaction while others did not. Although seven of the SARS-CoV-2 neutralizing mAbs bound SARS-CoV-1, only four mAbs neutralized both viruses. Three targeted the RBD, and one targeted the S2 subunit. Using the K18-hACE transgenic mouse model, therapeutic treatment with CV1-30, a potent RBD-binding Ab, reduced lung viral loads and protected mice from SARS-CoV-2 infection. In contrast, a weaker anti-RBD neutralizing mAbs, CV2-75, and the anti-NTD neutralizing mAb, CV1-1, displayed minimal protective efficacies. These observations strongly suggest that neutralization potency, along with Ab epitope specificity, regulates the *in vivo* protective potential of anti-SARS-CoV-2 Abs. Interestingly, the anti-S2 mAb, CV3-25, was the only one that was unaffected by mutations found in the recently emerged B.1.351 variant. These mAbs, especially CV3-25, can serve as starting points for the development of immunogens to elicit protective nAb responses against multiple coronaviruses.

## Results

### Serum Ab titers and neutralizing activities against SARS-CoV-2

**Figure 1:**
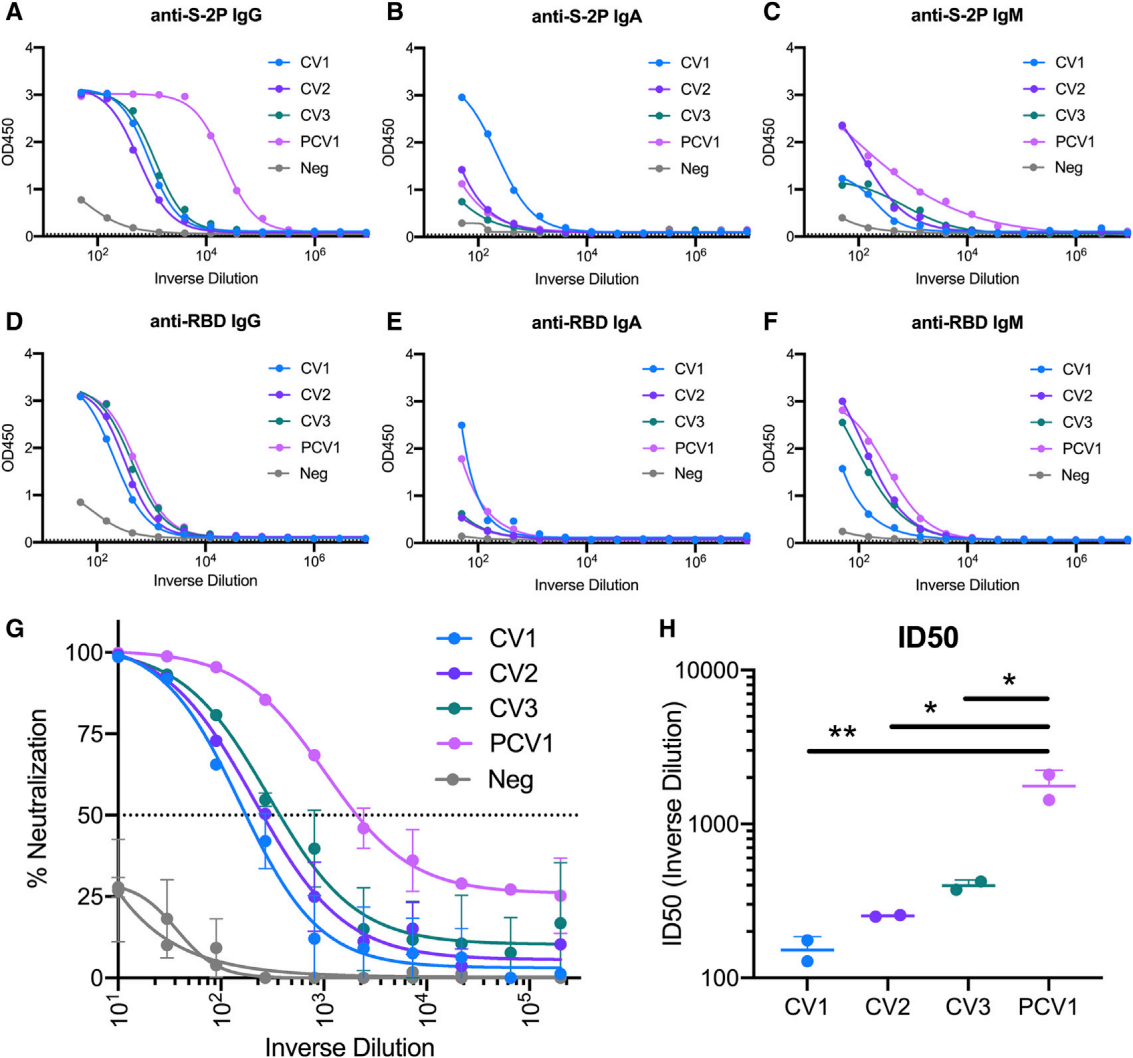
Serum Ab titers and neutralizing activities against SARS-CoV-2 Serum from four patients infected with SARS-CoV-2 (Table S1) was assessed for binding and neutralization capacity. (A–F) Serum Ab-binding titers to S-2P and the RBD were measured by ELISA in the four participants using the indicated isotype-specific secondary Abs. CV1: patient 1, collected 3 weeks post-symptom onset; CV2: patient 2, collected 3.5 weeks post-symptom onset; CV3: patient 3, collected 6 weeks post-symptom onset; PCV1: patient 4, collected 7 weeks post-symptom onset. Negative sera were collected prior to the SARS-CoV-2 pandemic. Dotted line indicates blank wells, the background reading. n = 2 ± standard deviation (SD). (G) Sera from the indicated donors were evaluated for their capacity to neutralize SARS-CoV-2 pseudovirus. n = 2 ± SD. (H) ID_50_ of serum neutralization. Values are shown for two independent replicates. Statistics evaluated as one-way ANOVA with Tukey’s multiple comparison test. n = 2 ± SD. Significance indicated for select comparisons. ^∗^p < 0.05, ^∗∗^p < 0.01, ^∗∗∗^p < 0.001, ^∗∗∗∗^p < 0.0001.

Peripheral blood mononuclear cells (PBMCs) and serum or plasma were collected from four SARS-CoV-2-infected adults (CV1 [previously discussed in Seydoux et al., 2020], CV2, CV3, and PCV1) at 3, 3.5, 6, and 7 weeks after the onset of symptoms, respectively (Table S1). Sera from PCV1 had the highest anti-stabilized spike (S-2P) immunoglobulin G (IgG) and IgM titers, while the anti-S-2P IgA titers were higher in CV1 (Figures 1A–1C). In contrast to the higher anti-S-2P IgG titers in the PCV1 sera, all four sera displayed similar anti-RBD IgG titers (Figures 1D–1F). PCV1 and CV1 had higher levels of anti-RBD IgA than did the other two donors, and CV1 showed slightly lower anti-RBD IgM than the three other sera.

While all sera neutralized SARS-CoV-2 (Figure 1G), serum from PCV1 was significantly more potent (Figure 1H). The serum neutralizing differences track with time point in infection, with the samples collected at later time points show greater potency, potentially indicating maturation of the humoral response. Thus, though all four patients had similar anti-RBD-binding Ab titers, PCV1 developed higher anti-S-2P-binding Ab titers and higher neutralizing titers than did the other three patients examined here.

### Specific variable region genes give rise to anti-S Abs during SARS-CoV-2 infection

mAbs have been isolated and characterized previously by us and others (Cao et al., 2020a; Ju et al., 2020; Kreer et al., 2020; Nielsen et al., 2020; Robbiani et al., 2020; Seydoux et al., 2020). We isolated individual S-2P+ and RBD+ IgG+ B cells (Table S1) from all four subjects. The percentage of S-2P+ cells in the four patients ranged from 0.23% to 1.84% of IgG+ B cells, of which 5%–12.7% targeted the RBD. In agreement with the above-discussed serum Ab observations, the frequency of S-2P+ IgG+ B cells in PCV1 was 3- to 8-fold higher than those in the other patients, while no major differences were observed in the frequencies of RBD+ IgG+ B cells among the four patients. As expected, the frequency of S-2P+ cells in a healthy (pre-pandemic) control individual (CN) was lower than those found in the four patients (0.104% and 0.128%), as was the frequency of RBD+ IgG+ B cells (first sort: 0.015%; second sort: 0.019%). A total of 341 heavy chains (HCs), 353 kappa light chains (κLCs), and 303 lambda light chains (λLCs) were successfully sequenced from the four SARS-CoV-2-positive donors (Table S1; Figure S1), from which 228 paired HC/LCs were generated, and 198 Abs were successfully produced and characterized. We isolated 59 paired mAb sequences from the healthy individuals and then successfully generated 36 mAbs. As discussed above, we performed an initial characterization of the 48 mAbs from CV1 (Seydoux et al., 2020). Here, we performed a more in-depth characterization of these mAbs.

**Figure 2:**
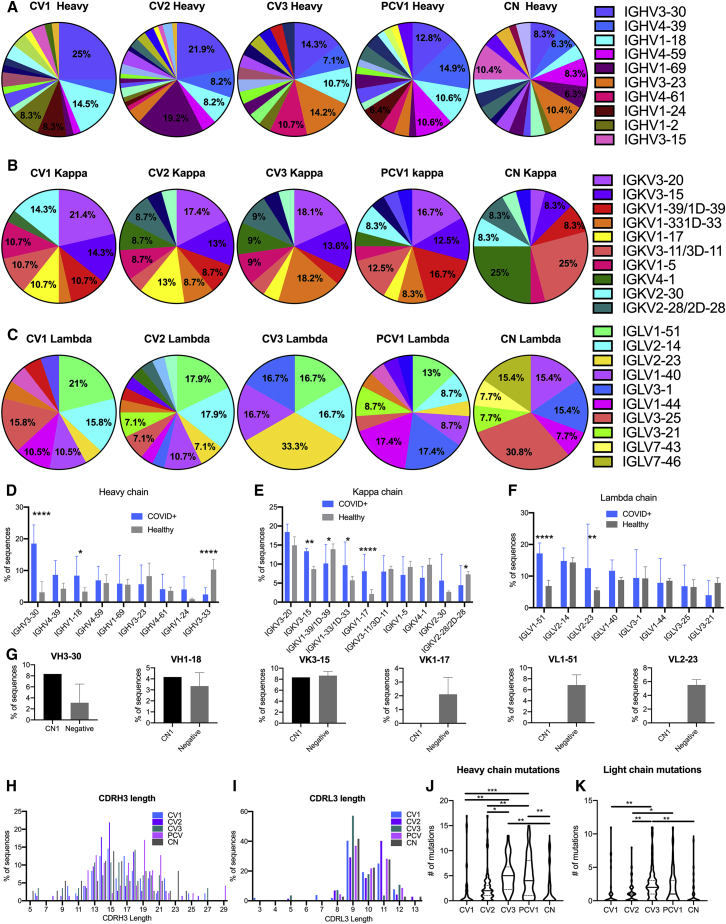
Specific VH and VL genes give rise to anti-S Abs during SARS-CoV-2 infection Sequences for the 198 mAbs elicited from the SARS-CoV-2-infected patients were compared for VH and VL gene usage. (A–C) The V gene usage was assigned for all paired heavy (A), kappa (B), and lambda (C) chains recovered from S-2P-specific B cells. Percentages are shown on graph for V chains that make up more than 5% of the total for each sort. Full sequencing data in Figure S1. (D–F) The frequency of select heavy (D), kappa (E), and lambda (F) chain V gene usage for the four COVID+, S-2P+ sorted participants is compared to five SARS-CoV-2-unexposed, “healthy” adult participants determined using unbiased 10× sequencing of total B cells. Error bars indicate standard deviation. Full sequencing in Figure S2A. (G) Comparison of VH3-30, VH1-18, VK3-15, CK1-17, VL1-51, and VL2-23 frequencies in S-2P+ sorted unexposed cells (CN) and B cells from five unexposed donors determined by unbiased sequencing (negative). Full sequencing in Figure S2B. (H and I) The CDR3 length distribution for the heavy (H) and light (I) chains shown as percentage of Abs from each donor. (J and K) The number of amino acid mutations in heavy (J) and light (K) chains of paired mAb sequences. Median is indicated as a solid line, with quartiles indicated in dashed lines. Significant differences were determined using one-way ANOVA with Tukey’s multiple comparison test ^∗^p < 0.05, ^∗∗^p < 0.01, ^∗∗∗^p < 0.001, ^∗∗∗∗^p < 0.0001.

In agreement with previous reports, the Abs isolated from the patients utilized diverse V regions (Cao et al., 2020a; Nielsen et al., 2020; Robbiani et al., 2020; Seydoux et al., 2020) (Figures 2A–2C and S1). Similarly, the S-specific mAbs isolated from the healthy donor originate from diverse V regions. To determine whether anti-S-2P+ B cells that express certain variable heavy (VH) and variable light (VL) genes preferentially expand during infection, we compared the relative frequencies of each VH and VL sequence to those present in healthy individuals. For this, we performed a 10×-based sequence analysis of total circulating B cells (i.e., not S-2P specific) from five SARS-CoV-2-unexposed adults (Figures 2D–2F and S2). Significantly higher frequencies of S-2P+ IGHV3-30 and IGHV1-18 Ab sequences were observed in the patients as compared to the relative frequencies of these two genes present in healthy adults (Figure 2D). Interestingly, lower frequencies of S-2P+ IGHV3-33 usage were observed in the patients than in healthy donors. Differences were also observed in kappa (Figure 2E) and lambda (Figure 2F) gene usage between patients and healthy donors. Specifically, IGKV3-15, IGKV1-33/1D-33, and IGKV1-17 were significantly elevated in patients as compared to healthy donors, while the expression of IGKV1-39/1D-39 was reduced. IGLV1-51 was significantly elevated in the patients as compared to healthy donors, as was IGLV2-23, though this appears to be driven by a greatly elevated usage in patient CV3.

The above observations suggest that naive B cell clones expressing the above IGHV, IGKV, or IGLV genes preferentially recognize the viral S protein at the initial stages of infection. To address this point, IgD+, IgM+, and S-2P+ B cells were isolated from CN (following two independent B cell sorting experiments from this donor) (Table S1); their V genes were sequenced; and their relative frequencies were again compared to those found in total B cells from healthy donors (Figure 2G). Although IGHV3-30 was present in higher frequency in B cells sorted with S-2P from CN than in the total B cell population, the difference was not as large as in the infected patients. Similarly, no differences were observed for the other IGHV and IGHV1-18, and no instances of the IGKV or IGLV genes that were predominant in the anti-S response after infection appeared in CN. Thus, it appears that the anti-spike B cell response that predominates at 3–7 weeks post-infection is dissimilar from the naive B cells that preferentially bind to S-2P.

The length distribution for the complementarity determining region H3 (CDR) H3 and CDR L3 of Abs was comparable to those present in the pre-infection, healthy B cell repertoires (Figures 2H and 2I). Interestingly, the IGHV and IGLV sequences derived from samples collected at 6 (CV3) and 7 (PCV1) weeks after infection had significantly more amino acid mutations than those derived from samples collected at 3 (CV1) or 3.5 (CV2) weeks after symptom development, or than the CN mAbs (Figures 2J and 2K). These observations are suggestive of a continuous B cell evolution during SARS-CoV-2 infection, as others recently reported (Gaebler et al., 2021).

### Epitope specificities and cross-reactivities of SARS-CoV-2 Abs

**Figure 3:**
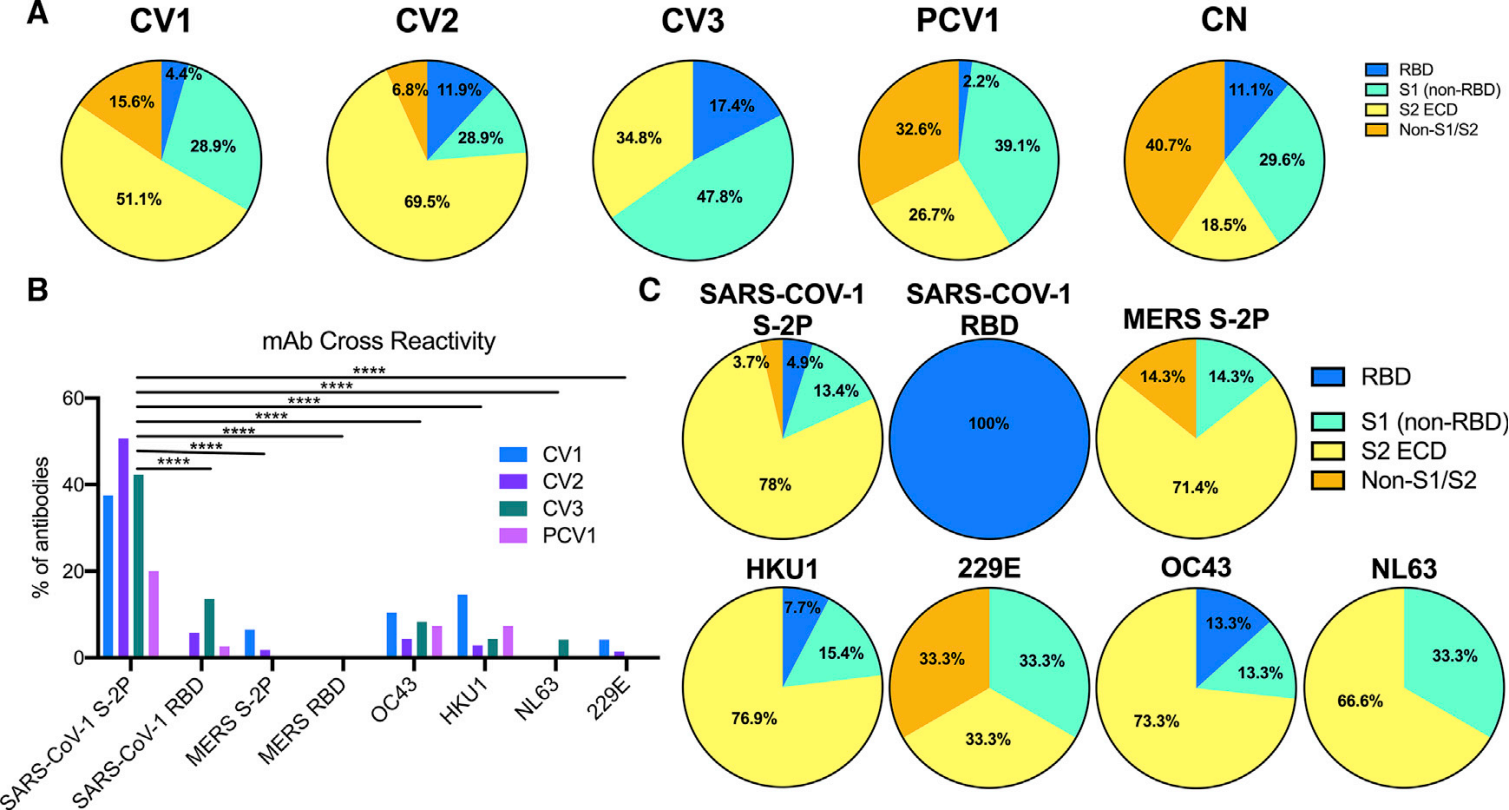
Epitope specificities and cross-reactivity of SARS-CoV-2 Abs The percentage of mAbs from each donor specific for the SARS-CoV-2 spike subdomains and their cross-reactivity was determined by biolayer interferometry (BLI). (A) Using S1 and S2 monomer proteins, mAbs were grouped into the Abs that bound the RBD in the S1 subunit (S1: RBD, blue), mAbs that bound S1 outside of the RBD (S1: non-RBD, teal), mAbs that bound the S2 ECD (S2 ECD, yellow), or those that bound S2P but did not bind either S1 or S2 (S2P: Non-S1/Non-S2). (B) The percentage of mAbs that bind to SARS-CoV-1, MERS, and the four common human coronavirus was also measured by BLI using S2-P timers for SARS-CoV1 and MERS and S1+S2 monomers for the four human coronavirus antigens. (C) The percentage of mAbs that bound each subdomain of the coronavirus spike for the mAbs cross-reactive with SARS-CoV-1, MERS S-2P, and the four common human coronaviruses. Only mAbs isolated from the four SARS-CoV-2-infected donors are included. Significant differences were determined using two-way ANOVA with Tukey’s multiple comparison test. ^∗^p < 0.05, ^∗∗^p < 0.01, ^∗∗∗^p < 0.001, ^∗∗∗∗^p < 0.0001. Additional BLI data and comparison to number of amino acid mutations in Figure S3.

The binding specificities of the 198 mAbs to S subdomains were determined using recombinant proteins including S1, RBD, N-terminal domain (NTD), and S2 ectodomain (ECD) monomer subunits (Figures 3A, S3A, and S3B). Only a small percentage of mAbs bound RBD, irrespective of the time of B cell isolation following the development of symptoms. However, the relative proportion of anti-S2 Abs was higher in samples collected at 3 and 3.5 weeks (51% in CV1 and 70% in CV2, respectively) than in samples collected 6 and 7 weeks post-symptom onset (35% in CV3 and 27% in PCV1, respectively). PCV1 had a high proportion (32%) of Abs whose epitopes could not be mapped to S1 or S2, while such Abs were rarer in the other three patients examined here (15% in CV1, 7% in CV2, and 0% in CV3). Out of the 36 mAbs produced from healthy individuals, 27 (75%) bound S2P, and of these, 40.7% could not be mapped to S1 or S2 binding.

We also determined the abilities of these Abs to recognize SARS-CoV-1; MERS; the two endemic human beta coronaviruses, OC43 and HKU1; and the two endemic human alpha coronaviruses, NL63 and 229E (Figure 3B). In total, 81 mAbs (41%) displayed SARS-CoV-1 reactivity (to varying degrees), approximately half of which recognized the SARS-CoV-1 RBD. In contrast, only 4 mAbs (2.3%) displayed cross-reactivity toward MERS (and none to the MERS RBD), 13 bound OC43 (7.1%), 12 bound HKU1 (6.6%), 2 bound NL63 (1.1%), and only 1 bound 229E (0.56%). Of these cross-reactive mAbs, the majority mapped to S2 binding (Figure 3C), and most bound only one coronavirus type beyond SARS-CoV-2 (Figure S3C). There was no association between the number of amino acid mutations in the Ab V regions and cross-reactivity with divergent HCoVs (Figures S3D–S3J).

### SARS-CoV-1 and SARS-CoV-2 cross-neutralizing properties of mAbs

**Figure 4:**
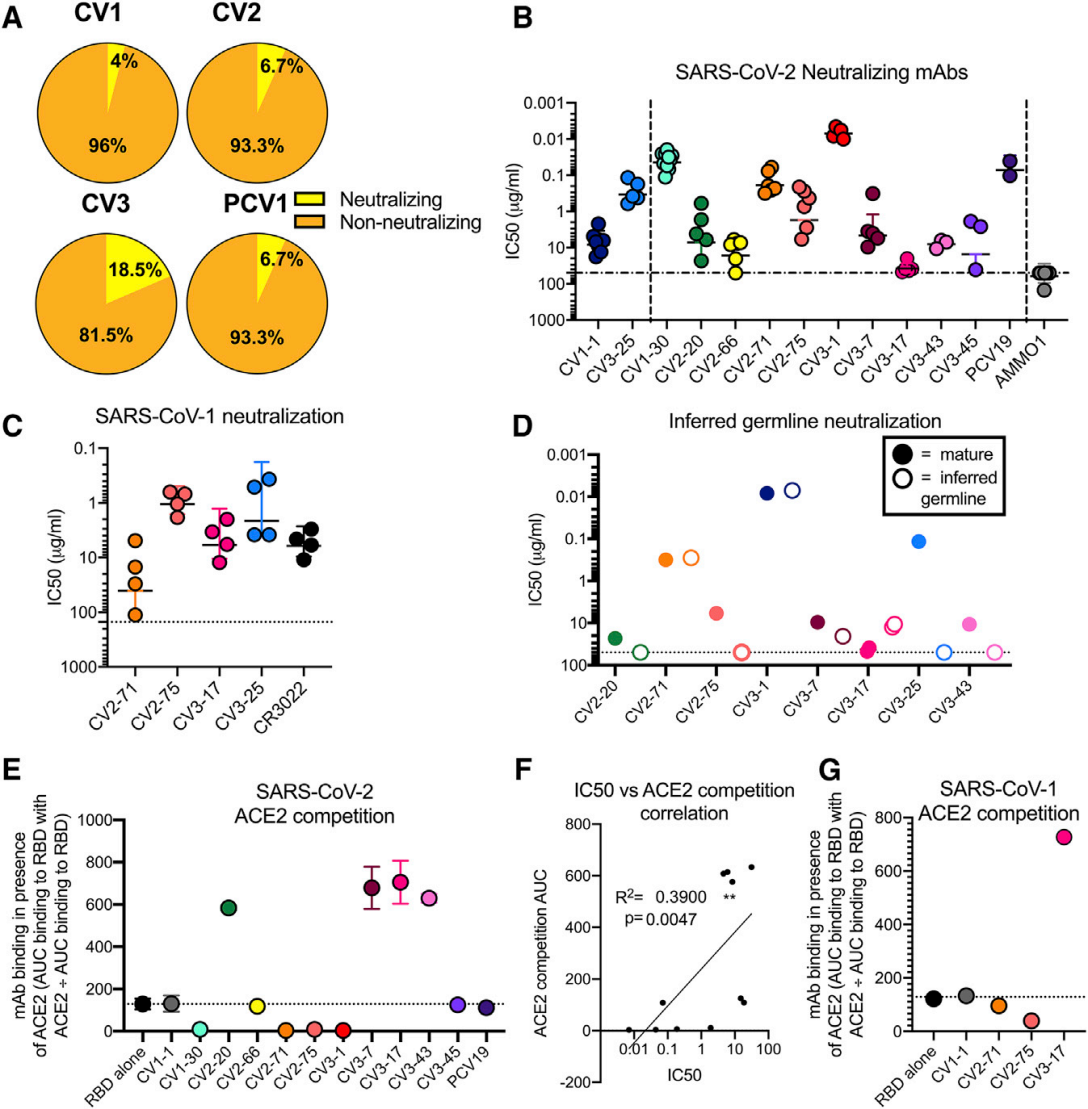
SARS-CoV-1 and SARS-COV-2 cross-neutralizing properties of mAbs The 14 neutralizing mAbs were characterized. (A) Percentage of mAbs capable of achieving 50% neutralization of SARS-CoV-2 pseudovirus at a concentration of 50 μg/ml from each donor. (B) The IC_50_s of each nAb in comparison to a negative control (AMMO1) are graphed. Each data point represents an independent replicate, with the mean and SD indicated with error bars. The non-RBD-binding mAbs, CV1-1 and CV3-25, on the left side of the graph are separated by a dashed line from the RBD-binding mAbs on the right side of graph. (C) SARS-CoV-2 neutralizing mAbs were assessed for their ability to neutralize SARS-CoV-1. CR3022 is a control SARS-CoV-1 neutralizing mAb. Horizontal line indicates mean with error bars at SD. Full data in Figures S4A–S4D. (D) The IC_50_s of the iGL versions of the mAbs (open dots) are compared to IC_50_s of mutated mAbs (solid dots). Additional data in Figure S5. (E) Competition of mAbs for binding to ACE2. mAbs that show competition have a binding signal below the dotted line and block ACE2 binding, and mAbs with a binding signal above the dotted line enhance ACE2 binding by increasing avidity through immune complex formation. Competition is calculated as the area under the curve (AUC) of mAb binding to the RBD in the presence of ACE2 divided by the AUC of mAb binding to RBD alone. Dots are shown as the median of two replicates, with SD indicated by error bars. The dotted line at the RBD-alone condition indicates BLI signal of uninhibited RBD:ACE2 binding. The NTD-specific CV1-1 mAb is used a negative control. (F) Correlation between SARS-CoV-2 neutralization IC_50_ with AUC of the BLI of competition with ACE2 for RBD binding. R^2^ value for nonlinear fit and Spearmen correlation p value are shown. (G) The competition of mAbs for binding to SARS-CoV-1 RBD with ACE2 is compared on this graph performed as in (E). Full ACE2 competition data in Figures S4F–S4J. Additional characterization of CV1-1 and CV2-75 in Figure S6.

**Figure 5:**
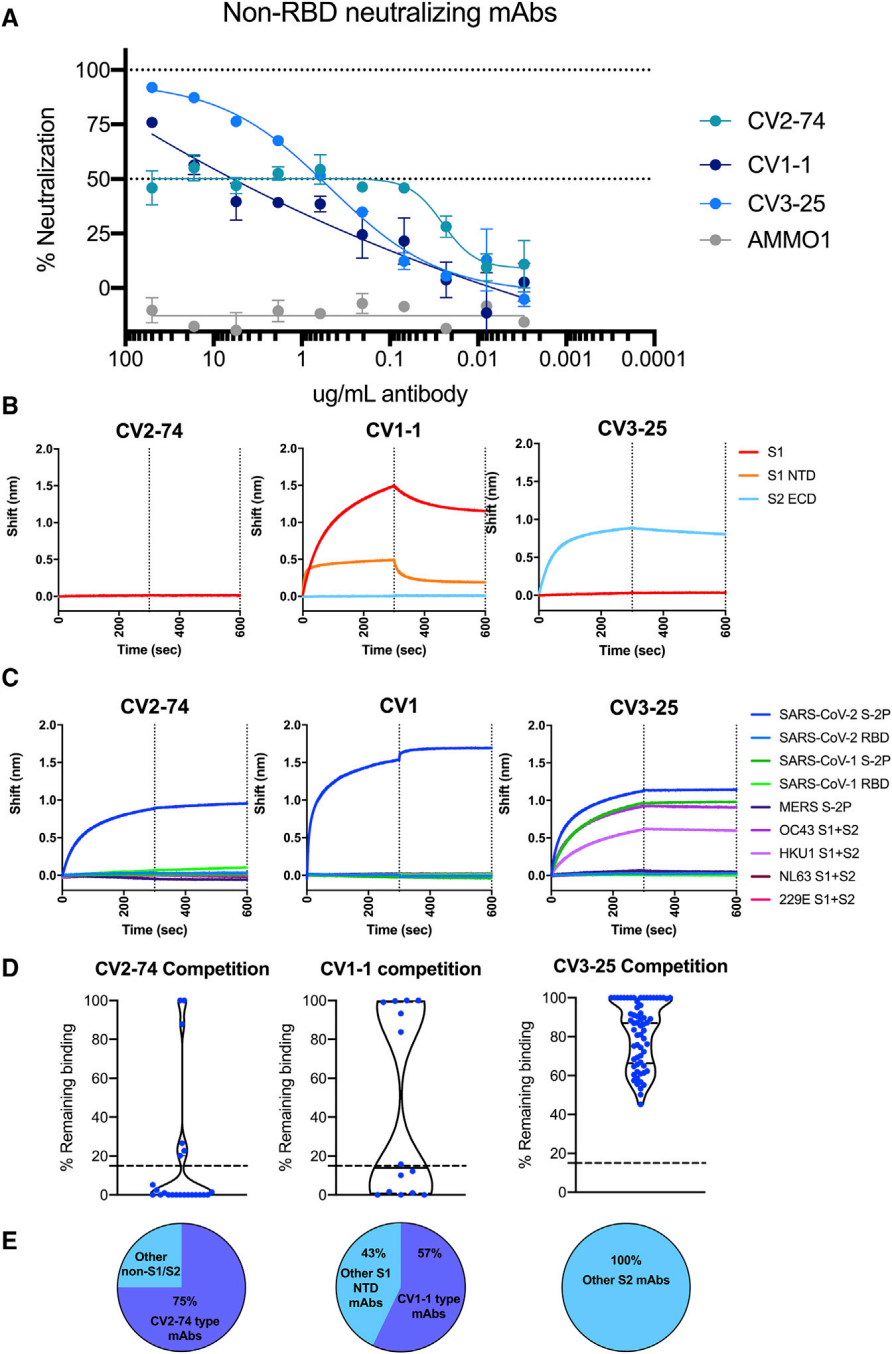
Neutralization by non-RBD-binding nAbs The three neutralizing, non-RBD-binding mAbs were characterized. (A) Neutralization curves for non-RBD-binding mAbs. Error bars indicate SD. (B) BLI traces for the indicated mAbs binding to SARS-CoV-2 S1 or S2 subunits or the NTD subdomain of S1. (C) BLI traces of mAbs incubated with human coronavirus antigens as indicated. (D) Violin plots show competitions between each non-RBD mAb and other mAbs. Each data point represents the AUC of an individual mAb binding to RBD (left), NTD (middle), or S2 (right) minus AUC of competition with either CV2-74 (left), CV1-1 (middle), or CV3-25 (right). Dotted line at 15% remaining binding indicates what is considered true competition, and dots below the line are considered competitive. For CV1-1, S1 NTD mAbs from CV1, CV2, and CV3 were tested. For CV2-74, all non-S1/S2 mAbs in all four sorts were tested. For CV3-25, all S2-binding mAbs in all four sorts were tested. Median of plot is indicated as a solid line, with quartiles indicated as dashed lines. (E) Pie charts show the percentage of mAbs in each set that effectively compete with each tested mAb. mAbs that competed are indicated in the purple section, while non-competitive mAbs are in blue.

Only 14 mAbs (7%) neutralized SARS-CoV-2 (Figure 4A), with IC_50_s ranging from 0.007 μg/ml to 15.1 μg/ml (although, as we discuss below, we were unable to assign an IC_50_ to CV2-74) (Figures 4B, 5A, and S4; Table S2). In total, 11 of 14 neutralizing mAbs bound RBD, in agreement with our recent report (Seydoux et al., 2020) and other reports that RBD is the major target of anti-SARS-CoV-2 nAbs (Barnes et al., 2020; Cao et al., 2020a; Ju et al., 2020; Liu et al., 2020; Rogers et al., 2020a). Three of the nAbs—CV1-1 (from patient CV1), CV2-74 (from patient CV2), and CV3-25 (from patient CV3)—bound epitopes outside the RBD. CV1-1 binds the S1 NTD, CV3-25 binds the S2 subunit, and CV2-74 bound neither the recombinant S1 or S2 proteins used here, and we were unable to define its specificity (Figures 5B, S4A, and S4B).

The three most potent nAbs, all anti-RBD, were CV1-30 (IC_50_ = 0.044 μg/ml) (Seydoux et al., 2020), CV3-1 (IC_50_ = 0.007 μg/ml), and PCV19 (IC_50_ = 0.072 μg/ml). The anti-NTD mAb (CV1-1) had lower neutralizing potency (IC_50_ = 8.2 μg/ml), and as we previously reported (Seydoux et al., 2020), its maximum level of neutralization was lower than 100% (Figure S4C), similar to other anti-NTD mAbs (Liu et al., 2020). CV1-1 displayed decreased binding against more stable SARS-2-CoV S-engineered proteins (S-6P), as shown by lower overall unit response and faster off-rate by biolayer interferometry (BLI), and does not bind like other published NTD-targeting Abs by negative-stain electron microscopy (EM) (Figures S5A and S5B) (Liu et al., 2020). The IC_50_ of anti-S2 mAb, CV3-25, was 0.34 μg/ml, which is comparable to most anti-RBD nAbs with the exception of CV1-30, CV3-1, and PCV19.

Out of the 14 nAbs, seven (CV2-20, CV2-71, CV2-75, CV3-7, CV3-17, CV3-25, and CV3-43) also bound the S-2P of SARS-CoV-1, and four of the seven neutralized this virus (Figures 4C and S4D; Table S2). Three were anti-RBD (CV2-71, CV2-75, and CV3-17), while the fourth, CV3-25, bound to S2 (Figure 5B; Table S2). Interestingly, while the IC_50_s of CV2-71, CV2-75, and CV3-25 against SARS-CoV-1 and SARS-CoV-2 were not significantly different, CV3-17 neutralized SARS-CoV-1 more potently than SARS-CoV-2 (Figure S4E). Furthermore, the two most potent anti-SARS-CoV-2 mAbs (CV1-30 and CV3-1) did not neutralize SARS-CoV-1.

### Neutralization of iGL forms of mAbs

CV1-30 has only two non-silent somatic mutations (both in VH) that we previously reported are important for potent neutralization of SARS-CoV-2 (Hurlburt et al., 2020). To examine if this is a general phenomenon among anti-RBD SARS-CoV-2 nAbs, we generated the inferred-germline (iGL) versions of six anti-RBD Abs (CV2-20, CV2-71, CV2-75, CV3-1, CV3-7, and CV3-43) and measured their neutralizing potencies (Figures 4D and S6A). Three of six anti-RBD iGL-nAbs—CV2-20 (three amino acid mutations), CV2-75 (three amino acid mutations), and CV3-43 (nine amino acid mutations)—were non-neutralizing. However, no differences in neutralizing potency between the mutated and iGL-CV2-71 (three amino acid mutations), iGL-CV3-1 (two amino acid mutations), and iGL-CV3-7 (nine amino acid mutations) were observed. Reductions in neutralizing potency of the iGL mAbs correlated with faster dissociation rates from the RBD (Figure S6B). The anti-NTD mAb CV1-1 has no amino acid mutations in its V genes, while the anti-S2 Ab CV3-25 has five mutations. Reversion of the anti-S2 mAb CV3-25 to its GL form also led to a significant reduction in its neutralizing potency. Thus, some anti-SARS-CoV-2 nAbs are capable of potent neutralization in the absence of affinity maturation, while the neutralizing activity of others depends on the accumulation of a small number of mutations. Overall, however, there was no correlation between the neutralization potency and the degree of somatic hypermutation (data not shown).

### Potent anti-RBD nAbs block the binding of ACE-2 to the RBD

We next examined whether the differences in neutralizing potencies of the anti-RBD nAbs (Figure 4B) were due to differences in their relative abilities to block the RBD-ACE2 interaction (Figures 4E and S4F). While CV2-71, CV2-75, and CV3-1 abolished ACE2 binding to the RBD—suggesting that they either directly bound the receptor-binding motif (RBM), like CV1-30 (Hurlburt et al., 2020), or indirectly (sterically) hindered this binding—the remaining seven anti-RBD NAbs (CV2-20, CV2-66, CV3-7, CV3-17, CV3-43, CV3-45, and PCV19) did not inhibit the RBD-ACE2 interaction. Similar observations were made when the abilities of mAbs to block the interaction of recombinant S-2P to cells expressing ACE2 were examined (Figure S4G). Indeed, a correlation between the potency of neutralization and the extent to which a mAb blocked the RBD-ACE2 interaction was observed (Figures 4F and S4H), in agreement with previous reports (Baum et al., 2020a; Brouwer et al., 2020; Gavor et al., 2020; Hoffmann et al., 2020; Wan et al., 2020a; Yan et al., 2020).

As mentioned above, three of the anti-RBD mAbs (CV2-71, CV2-75, and CV3-17) also neutralized SARS-CoV-1. The abilities of these Abs to block the ACE2 interaction with the SARS-CoV-1 RBD were similar to their abilities to block the interaction of ACE2 with the SARS-CoV-2 RBD, with CV2-71 and CV2-75 blocking ACE2 interaction to some degree (Figures 4G, S4I, and S4J). Abs like CV1-30 and CV3-1 that potently neutralize SARS-CoV-2 and block the interaction between SARS-CoV-2 RBD and ACE2 fail to mediate SARS-CoV-1 neutralization because they bind the RBM in the RBD, which has limited sequence homology, to that of SARS-CoV-1 RBD (Hurlburt et al., 2020). In contrast, CV2-75 binds the RBD at an epitope distinct from the RBM (Figure S5C; Table S3) and is only accessible when the RBD is in the up conformation. The residues that CV2-75 interacts with on the RBD are nearly completely conserved between SARS-CoV-1 and SARS-CoV-2, explaining the cross-neutralizing ability (Figure S5D). An alignment with the structure of ACE2-RBD showed that the heavy chain of CV2-75 would clash with the glycan at Asn322 in ACE2, establishing a mechanism of competition (Figure S5E).

### Neutralization by non-RBD-binding Abs

As mentioned above, CV2-74 binds to an undefined epitope on S that is present on S-2P but absent or not properly presented on the recombinant S1 or S2 proteins used here (Figure 5B). We identified several mAbs sharing this binding property (especially in PCV1), and the majority (75%) of these mAbs did compete the binding of CV2-74 to S-2P (Figures 5D and 5E). The fact that among these mAbs, only CV2-74 displayed neutralizing activity suggests that either the other mAbs bind distinct epitopes on S-2P and indirectly affect the binding of CV2-74 to S-2P or that CV2-74 binds a unique but overlapping epitope. It is noteworthy that CV2-74 displays an unusual neutralization curve, where the mAb neutralizes only 50% of the virus across a 1,000-fold concentration range (Figure 5A). For that reason, we did not assign an IC_50_ value to CV2-74.

Out of the 14 anti-NTD mAbs we identified, eight (57%) competed the binding of CV1-1 to S-2P (Figures 5D and 5E), and yet CV-1-1 was the only neutralizing anti-NTD mAb (Figure 4B). Interestingly, CV1-1 displayed decreased binding to more stable SARS-CoV-2-engineered soluble proteins (Figure S5). While BLI revealed binding of CV1-1 to recombinant NTD, the on-rate and maximal binding signal was lower than to the entire S1 domain, suggesting that secondary (or quaternary) contacts are important (Figure 5B). Indeed, negative-stain EM analysis indicates that it recognizes the NTD differently than other anti-NTD mAbs (such as COVA1-22; Brouwer et al., 2020), with a footprint that might also include an area just above the S1/S2 cleavage site (Figure S5B).

Out of 87 anti-S2 mAbs, CV3-25 was the only one capable of neutralizing SARS-CoV-2 and SARS-CoV-1 (Figures 4B and 4C) and of binding the S proteins of the OC43 and HKU1 betacoronaviruses (Figure 5C; Table S2). As none of the other 86 anti-S2 mAbs competed the binding of CV3-25 to S2-P (Figures 5D and 5E), we expect that CV3-25 binds a unique epitope on the S2 subunit, which is present not only on SARS-CoV-1, but also on the other coronaviruses tested here.

### Neutralizing mAbs as pre-exposure prophylaxis in k18-hACE2 mice

To assess whether nAbs with different epitope specificities offer the same level of protection *in vivo*, we compared the protective abilities of CV1-1, CV1-30, and CV2-75 in the K18-hACE2 mouse model (Winkler et al., 2020). As discussed above, CV1-1 binds the NTD and has an IC_50_ of 8.2 μg/ml, while CV1-30 and CV2-75 bind the RBD and have IC_50_s of 0.044 and 1.7 μg/ml, respectively. Thus, CV1-1 and CV2-75 have neutralizing potentials in a similar range but recognize different regions of the viral spike.

**Figure 6:**
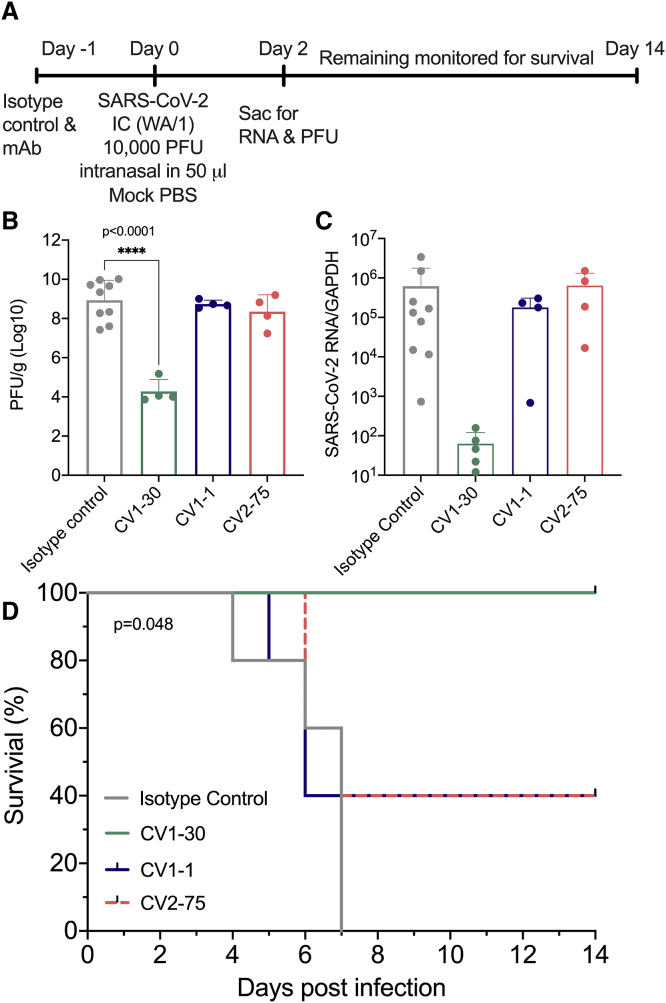
Neutralizing mAbs as pre-exposure prophylaxis in k18-hACE2 mice CV1-1, CV1-30, and CV2-75 were assessed to see whether they could confer protection in a mouse model. (A) Experimental timeline. (B) Number of PFUs in the lungs 2 days following challenge. Error bars indicate SD. (C) Viral RNA in lung tissue 2 days after challenge was measured by qPCR and normalized to GAPDH expression. Error bars indicate SD. (D) Kaplan-Meyer survival curve of the viral load/titer in the lungs of remaining mice comparing the various treatment groups. Statistics were determined by one-way ANOVA with Dunnett’s multiple comparison test. ^∗^p < 0.05, ^∗∗^p < 0.01, ^∗∗∗^p < 0.001, ^∗∗∗∗^p < 0.0001.

Mice were given a dose of 10 mg/kg of CV1-1, CV2-75, CV1-30, or an isotype control anti-Epstein-barr virus Ab, AMMO1 (Snijder et al., 2018), and then challenged intranasally with 10,000 plaque forming units (PFUs) of SARS-CoV-2 (Figure 6A). Two days post-challenge, half of the animals were euthanized to assess viral loads in the lung, and the remaining five animals were monitored for survival for up to 14 days. Two days post-challenge, mice receiving AMMO1, CV1-1, and CV2-75 had high levels (1 × 10^8^ PFU) of infectious virus and viral RNA in the lung (Figures 6B and 6C). Three of the five remaining animals in the CV1-1 and CV2-75 groups did not survive beyond 6 days post-challenge (Figures 6C and 6D). In contrast, CV1-30 significantly limited viral replication in the lungs at 2 days post-challenge (Figures 6B and 6C), and all remaining mice survived (Figure 6D).

Collectively, these data suggest that both neutralizing potency and epitope specificity are the most influential factors in defining the prophylactic efficacy of anti-SARS-CoV-2 Abs.

### Neutralization of the mutant SARS-CoV-2 B.1.351 variant

Recently, lineages of viral variants have emerged in the United Kingdom (B.1.1.7), South Africa (B.1.351), and Brazil (P.1) that harbor specific mutations in their S proteins that may be associated with increased transmissibility (Davies et al., 2020; Faria et al., 2021; Rambaut et al., 2020; Sabino et al., 2021; Tegally et al., 2020; Volz et al., 2021). The B.1.351 lineage appears to be more resistant to convalescent sera and mAbs (Edara et al., 2021; Liu et al., 2021; Stamatatos et al., 2021; Wang et al., 2020b; Wibmer et al., 2021; Wu et al., 2021). It is defined by several mutations in the RBD (K417N, E484K, N501Y), NTD (D80A, D215G,) and S2 (D614G) (O’Toole et al., 2021; Tegally et al., 2020). Other mutations are also found in the B.1.351 lineage in the NTD R246I and deletion 242–244 and S2 A701V, but at lower frequencies.

**Figure 7:**
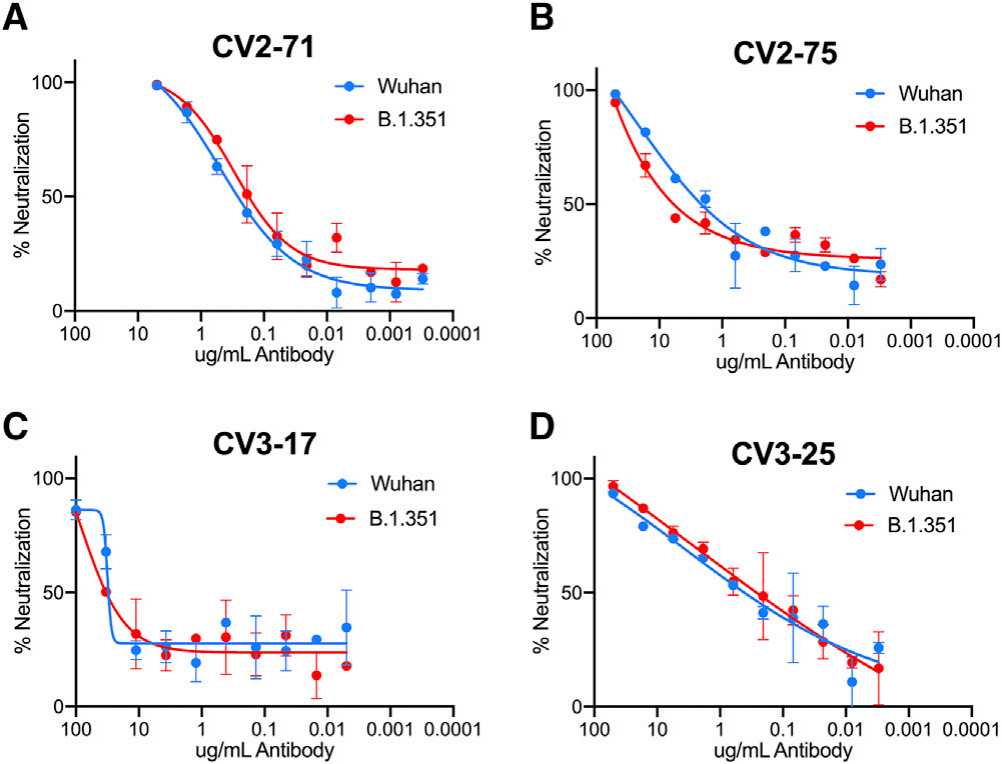
Neutralization of the mutant B.1.351 variant The SARS-CoV-1 neutralizing mAbs were tested against the B.1.351 strain. (A) CV2-71. (B) CV2-75. (C) CV3-17. (D) CV3-25. Graphs show neutralization curves for the Wuhan strain of SARS-CoV-2 in blue and the curve for the B.1.351 strain in red. Bars indicate SD.

We recently reported that these mutations abrogated the neutralizing activity of CV1-1 and reduced the neutralizing activities of the two most potent nAbs, CV1-30 and CV3-1, but not of CV2-75 (Stamatatos et al., 2021). Here, we evaluated the ability of the four cross-neutralizing mAbs (CV2-75, CV3-17, CV2-71, and CV3-25) to neutralize the B.1.351Δ242-243 mutant strain (Figure 7B) (Stamatatos et al., 2021). We found that all four mAbs retained their neutralizing activities against B.1.351.

## Discussion

Our study reveals that naive B cells expressing VH3-30 and VH1-18 preferentially recognize the SARS-CoV-2 envelope spike, but nAbs are produced by B cells expressing diverse B cell receptors (BCRs). Of the 198 mAbs characterized here (isolated at 3–7 weeks post-symptom development), 14 (7%) displayed neutralizing activities, and among them, only CV3-7 was derived from VH3-30. In fact, the 11 anti-RBD nAbs were derived from distinct B cell clones that cross-competed for binding, and four prevented the RBD-ACE2 interaction. These observations, combined with the fact that anti-RBD nAbs can neutralize the virus with no or minimal somatic mutation, may explain why potent anti-SARS-CoV-2 nAb responses are rapidly generated within a few weeks of infection or shortly following two immunizations with vaccines that express the viral spike (Jackson et al., 2020; Walsh et al., 2020). The observation that 7 of 11 anti-RBD nAbs do not prevent the RBD-ACE2 interaction indicates different mechanisms of neutralization by anti-RBD Abs. The former nAbs may prevent RBD-heparin interactions (Clausen et al., 2020), stabilize the RBDs in their “up” conformation, and thus prematurely activate the fusion machinery (Koenig et al., 2021; Wrapp et al., 2020a); or they may limit the conformational changes, and particularly the RBD movement, that are required for cell fusion, allowing them to neutralize without directly blocking ACE2 binding.

The two most potent anti-SARS-CoV-2 nAbs, CV1-30 and CV3-1—which both bind SARS-CoV-2 RBD but not SARS-CoV-1 RBD—did not neutralize SARS-CoV-1, while CV2-75 and CV3-17—which bind not only SARS-CoV-2 RBD, but also SARS-CoV-1 RBD and display weaker anti-SARS-CoV-2 neutralizing activities—were able to efficiently neutralize SARS-CoV-1. A comparison of the CV2-75-RBD and CV1-30-RBD (Hurlburt et al., 2020) structures reveals that CV2-75 binds an area of SARS-CoV-2 RBD with higher sequence homology with SARS-CoV-1 RBD. In contrast, CV1-30 binds directly to the RBM, which only has 50% sequence homology among SARS-CoV-1 and SARS-CoV-2 (Finkelstein et al., 2021; Hurlburt et al., 2020; Wan et al., 2020b).

The mechanisms of neutralization of the three non-RBD-binding nAbs characterized here (CV1-1, CV2-74, and CV3-25) are presently unknown. As CV1-1, CV2-74, and CV3-25 do not interfere with the binding of ACE-2 to S-2P, we anticipate that they mediate neutralization by interfering with a step in the fusion process that follows attachment. The viral spike undergoes conformational changes, specifically in the S2 region, during virus-cell binding and fusion (Cai et al., 2020; Gavor et al., 2020; Walls et al., 2020). These three mAbs may prevent these conformational changes from occurring, either by locking the spike in an intermediate conformation, preventing cleavage, or stabilizing its pre-fusion conformation.

The fact that the binding of CV1-1 to S-2P was competed by the other anti-NTD mAbs (14 total), all of which were non-neutralizing, suggests that CV1-1 recognizes the NTD in a distinct manner from the non-neutralizing anti-NTD mAbs. Similarly, the binding of CV2-74 to S-2P was competed by the other non-neutralizing non-S1/S2 mAbs (23 total), which strongly suggests that these mAbs all recognize the same immunogenic region, but CV2-74 recognizes it in a unique manner. In contrast, none of the anti-S2 mAbs isolated here (65 total) competed the binding of CV3-25 to S-2P. These observations and the fact that CV3-25 potently neutralizes both SARS-CoV-1 (IC_50_ 2.1 μg/mL) and SARS-CoV-2 (IC_50_ 0.34 μg/mL) and the B.1.351 mutant strain and binds the S proteins of HKU1 and OC43 strongly suggests that it recognizes a conserved epitope among diverse coronaviruses. As only two other anti-S2 Abs that neutralize both SARS-CoV-1 and SARS-CoV-2 (but with weaker neutralizing activities than CV3-25) were reported so far (Song et al., 2020; Wang et al., 2020b), we expect the epitope of CV3-25 to be less immunogenic than those recognized by non-neutralizing anti-S2 Abs.

We propose that because of its cross-neutralizing activity and its ability to neutralize the B.1.351, and because it binds the OC43 and HKU1 spikes, CV3-25 is a potential starting point for developing a pan-coronavirus vaccine. We expect that the protective potentials of Abs that bind the same region of S2 as CV3-25 could be improved through the accumulation of amino acid mutations in their VH and VLs by sequential immunizations. As a first step, the epitope of CV3-25 must be identified, and immunogens should be designed expressing it in the most immunogenic form.

In summary, our study indicates that neutralization of SARS-CoV-2 and SARS-CoV-1 does not necessitate the expansion of B cell lineages that express particular VH/VL pairings and that even the unmutated forms of some Abs can potently neutralize SARS-CoV-2 and SARS-CoV-1. As these viruses are capable of tolerating mutations in distinct regions of its viral spike, they will be able to escape the neutralizing activities of most nAbs. The S2 subunit, however, contains at least one epitope that, although poorly immunogenic, is present on four of five human beta coronaviruses. That epitope, as defined by its recognition by CV3-25, is a valid candidate for the development of a global coronavirus vaccine.

## STAR★Methods

### Key resources table

**Table:** 

REAGENT or RESOURCE	SOURCE	IDENTIFIER
**Antibodies**		

CD14 PE-Cy5	eBiosciences	Cat# 15-0149-42; RRID:AB_2573058
CD69 APC-Fire750	Biolegend	Cat# 310946; RRID:AB_396953
CD8a Alexa Fluor 700	BD Biosciences	Cat# 557945; RRID:AB_396953
CD3 BV510	Biolegend	Cat# 317332; RRID:AB_396953
CD27 BV605	Biolegend	Cat# 302830; RRID:AB_2561450
IgM PE-Dazzle594	Biolegend	Cat# 314530; RRID:AB_2566483
CD4 BB515	BD Biosciences	Cat# 565996; RRID:AB_2739447
IgD BV650	BD Biosciences	Cat# 740594; RRID:AB_2740295
IgG BV786	BD Biosciences	Cat# 564230; RRID:AB_2738684
CD56 PE-Cy5	BD Biosciences	Cat# 318308; RRID:AB_604105
CD19 PE-Cy7	BD Biosciences	Cat# 557835; RRID:AB_396893
CD38 PerCP-Cy5.5	BD Biosciences	Cat# 561106, RRID:AB_2033958
Goat anti-human IgG HRP	Southern Biotech	Cat# 2010-05, RRID:AB_2795564
Goat anti-human IgA HRP	Southern Biotech	Cat# 2050-05, RRID:AB_2687526
Goat anti-human IgM HRP	Southern Biotech	Cat# 2020-05, RRID:AB_2795603
AMMO1	Snijder et al., 2018	N/A

**Bacterial and virus strains**		

OneShot DH5 Alpha cells	Thermo Fisher Scientific	Cat#: 12297016
icSARS-CoV-2 virus	Xie et al., 2020	N/A

**Biological samples**		

PBMC from SARS-CoV-2 infected donots	This study	N/A
Sera from SARS-CoV-2 infected donors	This study	N/A
PBMC from pre-pandemic donors	This study	N/A
Sera from pre-pandemic donors	This study	N/A

**Chemicals, peptides, and recombinant proteins**		

polyethyleneimine	Polyscience	Cat#24765
HisTrap FF affinity column	GE Healthcare	Cat# 17-5255-01
StrepTactin Sepharose column	IBA Lifesciences	Cat# 2-1201-002
Superose 6 10/300 GL column	GE Healthcare	Cat# 17-5172-01
HiLoad 16/600 Superdex 200 pg column	GE Healthcare	GE28-9893-36
Strep-Tactin Purification Buffer Set	IBA Lifesciences	Cat#2-1002-001
protein A agarose resin	Goldbio	Cat# P-400
HRV3C protease	This study	N/A
HCoV-OC43	Sino biologics	40607-V08B
HCoV-HKU1	Sino biologics	40606-V08B
HCoV-NL63	Sino biologics	40604-V08B
HCoV-229E	Sino biologics	40605-V08D
SARS-HCoV-2 S1	Sino biologics	40591-V08B1
SARS-CoV-2 S1 N-terminal domain	Sino biologics	Cat#40591-V41H
SARS-HCoV-2 S2 extra-cellular domain	Sino biologics	Cat#: 40590-V08B
SARS-CoV2 RBD	Sino biologics	Cat#: 40150-V05H
SARS-CoV-1 RBD	Sino biologics	Cat#: 40150-V08B2
SureBlue TMB Microwell Peroxidase Substrate	Seracare KPL	Cat#: 5120-0075
Easylink NHS-biotin kit	Thermo Fisher Scientific	Cat#: 21425
Enrich SEC 650 10 × 300 mm column	BioRad	Cat#: 7801650
Streptavidin-Phycoerythrin (PE)	Invitrogen	Cat#: S21388
Streptavidin-BV711	Biolegend	Cat#: 405241
Alexa Fluor 647-labeled streptavidin	Invitrogen	Cat#: S32357
7AAD (7-Aminoactinomycin D)	Invitrogen	Cat#: A1310
iScript	Bio-Rad	Cat#: 1708891
HotStarTaq Plus	Quiagen	Cat#: 203607
GeneAmp dNTP Blend	ThermoFisher Scientific	Cat#: N8080261
Gel Red Nucleic Acid Stain	Biotium	Cat#: 41002
ExoSAP-IT	Affymetrix	Cat#: 78201
Exonuclease I	NEB	Cat#: M0293S
rAPid Alkaline Phosphatase	Sigma	Cat#: 4898133001
InFusion cloning	InFusion HD Cloning Kit	Cat#: 639649
Platinum SuperFi II DNA polymerase	Invitrogen	Cat#: 12358010
dpnI	NEB	Cat#: R0176S
PCR clean-up kit	NEB	Cat#: T1030S
QIAprep Spin Miniprep Kit	QIAGEN	Cat#: 27106
Freestyle 293 media	ThermoFisher Scientific	Cat#: 12338018
Protein A agarose beads	Thermofisher	Cat#: 20334
EasySep Human B cell Isolation Kit	Stem Cell Technologies	Cat #: 17954
Chromium Single Cell 5′ Library and Gel Bead Kit	10X Genomics	Cat#: 1000014
Chromium Single Cell A Chip Kit	10X Genomics	Cat#: 1000009
Human B cell Chromium Single Cell V(D)J Enrichment Kit	10X Genomics	Cat#: 1000016
EZ-Link NHS-PEG4-Biotin	Thermofisher Scientific	*Cat#: 21362*
293 Free transfection reagent	EMD Millipore	Cat#: 72181
Steady-Glo luciferase reagent	Promega	Cat# E2550
DY-549-labeled strep-tactin	IBA lifesciences	Cat #2-1565-050
Allphycocyanin-labeled streptavidin	Agilent	Cat #PJ27S-1
LysC	NEB	Cat# P8109S
MCSG Suite	Anatrace	Cat#s: MCGS-1T, MCGS-2T, MCGS-3T, MCGS-4T
Additive Screen	Hampton Research	Cat#: HR2-138
Tri Reagent	Zymo	Cat#: R2050-1-200
Direct-zol RNA MiniPrep Kit	Zymo	Cat#: R2051
High-capacity Reverse Transcriptase cDNA Kit	Thermo Fisher Scientific	Cat#: 4368813
Prime Time Gene Expression Master Mix	IDT	Cat#: 1055770

**Deposited data**		

CV1 H, K and L chain sequences	GenBank	GenBank: MT462477 - GenBank: MT462570
CV2 H, K and L chain sequences	GenBank	GenBank: MW681614 - GenBank: MW681759
CV3 H, K and L chain sequences	GenBank	GenBank: MW681558 - GenBank: MW681613
PCV H, K and L chain sequences	GenBank	GenBank: MW806097 - GenBank: MW806188
CN H, K and L chain sequences	GenBank	GenBank: MZ151189 - GenBank: MZ151260
CV2-75 Fab bound to SASR-CoV-2 RBD	This paper	PDB: 7M3I
CV30 Fab bound to SARS-CoV-2 RBD	Hurlburt et al., 2020	PDB: 6XE1
Germline PGT121 Fab structure	Mouquet et al., 2012	PDB: 4FQQ

**Experimental models: Cell lines**		

HEK293-EBNA1-6E	National Research Council, Canada	RRID:CVCL_HF20
HEK293T cells	ATCC	RRID:CVCL_0063
HEK293T-hACE2	BEI Resources	NR-52511
Vero-E6 hACE2/TMPRSS2	ATCC	ATCC Cat# CRL-1586, RRID:CVCL_0574

**Experimental models: Organisms/strains**		

B6.Cg-Tg(K18-ACE2)2Prlmn/J	Jackson Labs	JAX:034860, RRID:IMSR_JAX:034860

**Oligonucleotides**		

Primers for antibody nested PCR and sequencing	Tiller et al., 2008	N/A

**Recombinant DNA**		

pαH-SARS-CoV-2 S-2P	Wrapp et al., 2020b	N/A
pαH-SARS-CoV S-2P	Pallesen et al., 2017	N/A
pαH-MERS S-2P	Pallesen et al., 2017	N/A
pαH-RBD-Fc	Wrapp et al., 2020b	N/A
pαH-SARS-CoV RBD-Fc	Pallesen et al., 2017	N/A
pαH-MERS RBD-Fc	Pallesen et al., 2017	N/A
pTT3 IgL and IgL expression vectors	Snijder et al., 2018	N/A
pT4-341 HC vector	Mouquet et al., 2010	N.A.
pHDM-Hgpm2	BEI Resources	Cat# NR-52517
pRC-CMV-rev1b	BEI Resources	Cat# NR-52519
pHDM-tat1b	BEI Resources	Cat# NR-52518
pHDM-SARS-CoV-2 Spike	BEI Resources	Cat# NR-52514
pHAGE-CMV-Luc2-IRES-ZsGreen-W	BEI Resources	Cat# NR-52516
pHDM-SARS-CoV-2-SpikeB.1.351Δ242-243	Stamatatos et al., 2021	N/A
pHDM-SARS-CoV-1 Spike	Stamatatos et al., 2021	N/A
pαH-SARS-CoV-2 S-6P	Hsieh et al., 2020	N/A
Taqman Primer/Probe sets	IDT	Gapdh (Mm99999915_g1)

**Software and algorithms**		

Loupe V(D)J Browser (v. 3.0.0)	10X genomics	https://support.10xgenomics.com/single-cell-vdj/software/visualization/latest/installation
XDS	Kabsch, 2010	https://xds.mr.mpg.de/
Phaser	McCoy et al., 2007	https://www-structmed.cimr.cam.ac.uk/phaser_obsolete/
COOT	Emsley and Cowtan, 2004	https://www2.mrc-lmb.cam.ac.uk/personal/pemsley/coot/
Phenix	Adams et al., 2004	https://phenix-online.org/
Pymol	Schrödinger	https://pymol.org/2/
Leginon	Suloway et al., 2005	https://emg.nysbc.org/redmine/projects/leginon/wiki/Leginon_Homepage
Appion	Lander et al., 2009	https://emg.nysbc.org/redmine/projects/appion/wiki/Appion_Home
DogPicker	Voss et al., 2009	http://nramm.scripps.edu/software/dogpicker/
RELION 3.0	Scheres, 2012	https://www3.mrc-lmb.cam.ac.uk/relion/index.php/Main_Page
Geneious software (Version 8.1.9)	Geneious	https://www.geneious.com/
V Quest	Brochet et al., 2008	http://www.imgt.org/IMGT_vquest/user_guide
Prism	Graphad	https://www.graphpad.com/scientific-software/prism/
Flow Jo version 9.9.4	Tree Star	https://www.flowjo.com
ForteBio data analysis software	ForteBio	N/A

**Other**		

Biotek 405 select plate washer	BioTek	405 TS Washer
384-well nunclon plates	Thermo Fisher Scientific	Cat#: 164730
FACS Aria II	BD Biosciences	N/A
0.22 μM filter	ThermoFisher Scientific	Cat#: SE1M179M6
Amicon centrifugal filter	Thermo Fisher Scientific	Cat#: UFC901024
Octet Red 96e	ForteBio	N/A
Anti-Human IgG Fc capture (AHC) biosensors	Fortebio	Cat#18-5060
EZ-Link NHS-PEG4-Biotin	Thermo Fisher Scientific	CAT#: 21330
Streptavidin biosensors	Forte Bio	Cat#18-5019
Fluoroskan Ascent Fluorimeter	Thermofisher	Cat# 2805630
NT8 drop setter	Formulatrix	N/A
Tecnai Spirit (120 kV) microscope	Thermofisher	N/A
Omni-Bead ruptor tubes	VWR	Cat#: 10032-358
Omni Bead Ruptor 24	VWR	Cat#: 76000-746
QuantStudio5 qPCR system	Thermofisher	*Cat#: A34322*

### Resource availability

#### Lead contact

Further information and requests for resources and reagents should be directed to and will be fulfilled by the lead contact, Leonidas Stamatatos (lstamata@fredhutch.org)

#### Materials availability

This study did not generate new unique reagents

#### Data and code availability

The sequences for all mAbs isolated in this study have been uploaded to GenBank; CV1: GenBank: MT462477 - GenBank: MT462570; CV2: GenBank: MW681614 - GenBank: MW681759; CV3: GenBank: MW681558 - GenBank: MW681613; PCV: GenBank: MW806097 - GenBank: MW806188; and CN: MZ151189 - MZ151260. The structure of CV2-75 Fab bound to SARS-CoV-2 RBD has been uploaded to the Protein Databank. PDB: 7M3I. Other data will be made available upon request.

### Experimental model and subject details

#### Human subjects

Blood and peripheral blood mononuclear cells (PBMCs) were isolated from COVID19+ patients using protocols approved by Institutional Review Boards at Fred Hutch Cancer Research Center, University of Washington and Seattle Children’s Research Institute. PBMCs and serum from pre-pandemic controls were blindly selected at random from the study “Establishing Immunologic Assays for Determining HIV-1 Prevention and Control,” with no considerations made for age, or sex, participants were recruited at the Seattle Vaccine Trials Unit (Seattle, Washington, USA). Informed consent was obtained from all participants and the University of Washington and/or Fred Hutchinson Cancer Research Center and CHUM Institutional Review Boards approved the entire study and procedures.

#### Animal subjects

B6.Cg-Tg(K18-ACE2)2Prlmn/J mice were purchased from Jackson Laboratories. All mice used in these experiments were females between 8 −12 weeks of age. Mice were anesthetized with isoflurane and infected intranasally with SARS-CoV-2 in an ABSL-3 facility. Mice were monitored daily for weight loss. All experiments adhered to the guidelines approved by the Emory University Institutional Animal Care and Committee.

#### Cell lines

293-6E (human female) and 293T cells (human female) cells were maintained in Freestyle 293 media with gentle shaking. HEK293T-hACE2 (human female) were maintained in DMEM containing 10% FBS, 2 mM L-glutamine, 100 U/ml penicillin, and 100 μg/ml streptomycin (cDMEM). Vero-hACE2/TMPRSS2 (green monkey, female) were maintained in RPMI supplemented with 10% fetal bovine serum (FBS), 10 mM HEPES pH 7.3, 1 mM sodium pyruvate, 1 × non-essential amino acids, and 100 U/ml of penicillin–streptomycin. All cell lines were incubated at 37°C in the presence of 5% CO2.

### Method details

#### Recombinant proteins

pαH-derived plasmids encoding a stabilized His- and strep-tagged SARS-CoV-2 330 ectodomain (pαH-SARS-CoV-2 S-2P), SARS-CoV-2 S-6P (pαH-SARS-CoV-2 S-6P), SARS-CoV-1 S-2P (pαH-SARS-CoV S-2P), MERS S2-P (pαH-MERS S-2P), SARS-CoV-2 receptor binding domain (RBD) fused to a monomeric Fc (pαH-RBD-Fc), SARS-CoV-1 RBD fused to a monomeric Fc (pαH-SARS-CoV RBD-Fc) and MERS RBD (pαH-MERS RBD-Fc) fused to a monomeric Fc have been previously described and were a kind gift from Dr. Jason McLellan (Hsieh et al., 2020; Pallesen et al., 2017; Wrapp et al., 2020b).

Proteins were produced as described in Seydoux et al. (2020). Briefly, 1L of 293 EBNA cells at 1 × 10^6^ cells/mL were transfected with 500 mg of pαH-SARS-CoV-2 S2P, pαH-SARS-CoV S2P, pαH-SARS-CoV-2 RBD-Fc, pαH-SARS-CoV RBD-Fc, pαH-MERS S2P, pαH-MERS RBD-Fc using 2 mg of polyethyleneimine. After 6 days of growth, supernatants were harvested and filtered through a 0.22 mM filter. S2P supernatants were passed over a HisTrap FF affinity column and further purified using a 2 mL StrepTactin Sepharose column and a Strep-Tactin Purification Buffer Set. The S-2P variants were further purified using a Superose 6 10/300 GL column. RBD proteins were purified using protein A agarose resin, followed by on-column cleavage with HRV3C protease to release the RBD from the Fc domain. The RBD containing flow through was further purified by SEC using a HiLoad 16/600 Superdex 200 pg column. Proteins were flash frozen and stored at −80°C until use.

HCoV-OC43, HCoV-HKU1, HCoV-NL63 and HCoV-229E S1+S2 ECTs, SARS-HCoV-2 S1 domain, SARS-CoV-2 S1 N-terminal domain, SARS-HCoV-2 S2 extra-cellular domain (CAT#: 40590-V08B) and SARS-CoV-1 RBD were purchased from SinoBiologicals.

#### ELISA

S-2P and RBD were coated onto 384-well nunclon plates at 0.5 mg/mL in 30 mL overnight at 4C. Plates were washed with PBS 0.02% Tween (wash buffer) using a Biotek 405 select plate washer and then blocked in 100 mL of 10% milk, 0.02% Tween (Blocking/Dilution buffer) for 1 hour at 37C. Plates were washed again, and sera was loaded at a starting dilution of 1:50 with 11 serial 1:3 dilutions in dilution buffer in a total volume of 30 mL. After another hour at 37C, plates were washed again, and IgG, IgA or IgM was detected with 30 mL of HRP secondary (Goat anti-human IgG HRP, Goat anti-human IgA HRP, Goat anti-human IgM HRP) at a 1:3000 dilution for 1 hour at 37C. After the last wash, plates were developed with 30 mL SureBlue TMB Microwell Peroxidase Substrate. The reaction was quenched with 30 mL of 1N sulfuric acid. Plates were read on a SpectraMax M2 plate reader at 450 nM.

#### B cell sorting

B cell sorting was performed as described in Seydoux et al. (2020). Briefly, fluorescent probes were made from SARS-CoV-2 S-2P and RBD. S-2P and RBD were biotinylated protein at a theoretical 1:1 ration using the Easylink NHS-biotin kit according to manufacturer’s instructions. Excess biotin was removed via size exclusion chromatography using an Enrich SEC 650 10 × 300 mm column. The S-2P probes were made at a ratio of 2 moles of trimer to 1 mole streptavidin, one labeled with streptavidin-phycoerythrin (PE), and one with streptavidin-brilliant violent (BV) 711, both probes were used in order to increase the specificity of detection and reduce identification of non-specific B cells. The RBD probe was prepared at a molar ratio of 4 moles of protein to 1 mole of Alexa Fluor 647-labeled streptavidin. PBMCs from the five participants were thawed and stained for SARS-CoV-2-specific IgG+ memory B cells. First, cells were stained with the three SARS-CoV-2 probes for 30 minutes at 4°C, then washed, and stained with: viability dye (7AAD), CD14 PE-Cy5, CD69 APC-Fire750, CD8a Alexa Fluor 700, CD3 BV510, CD27 BV605, IgM PE-Dazzle594, CD4 brilliant blue 515 (BB515), IgD BV650, IgG BV786, CD56 PE-Cy5, CD19 PE-Cy7, and CD38 PerCP-Cy5.5 for another 30 minutes at 4°C. The cells were washed twice and resuspended for sorting in 10% FBS/RPMI containing 7AAD. Cells were sorted on a FACS Aria II by gating on singlets, lymphocytes, live, CD3-, CD14-, CD4-, CD19+, IgD-, IgG+, S-2P-PE+ and S-2P-BV711+. 10-18 million PBMCs were sorted from each participant with 384-1736 S-2P++ B cells sorted (Table S1). Cells were sorted into 96-well plates containing 16 μL lysis buffer (3.90% IGEPAL, 7.81 mM DTT, 1250 units/ml RNase Out).

#### PCR amplification and sequencing of VH and VL genes

RNA was reverse transcribed to cDNA by adding 4 ul of iScript to sorted B cells and cycling according to the manufacturer’s instructions. VH and VL genes were amplified using two rounds of PCR as previously described (Tiller et al., 2008). First round reactions contained 5 ul cDNA, 1-unit HotStarTaq Plus, 190 nM 3′ primer pool, 290 nM 5′ primer pool, 300 μM GeneAmp dNTP Blend, 2 ul 10x buffer, and 12.4 ul nuclease-free H2O. Second round PCR reactions used 5 ul first round PCR as template and 190 nM of both 5′ and 3′ primers. Second round PCR products were subjected to electrophoresis on a 1.5% agarose gel containing 0.1% Gel Red Nucleic Acid Stain. Positive wells were then purified using either ExoSAP-IT following manufacturer’s instructions or using a homemade enzyme mix of 0.5 units Exonuclease I, 0.25 units of rAPid Alkaline Phosphatase, and 9.725 ul 1x PCR buffer mixed with 5 ul of second round PCR product and cycled for 30 minutes at 37C followed by 5 minutes at 95C. Purified samples were Sanger sequenced. IMGT/V-QUEST was used to assign V, D, J gene identity, and CDRL3 length to the sequences (Brochet et al., 2008). Sequences were included in analysis if V and J gene identity could be assigned and the CDR3 was in-frame.

#### VH and VL cloning and antibody production

For sorts CV1, CV2 and CN, paired VH and VL sequences were optimized for human expression using the Integrated DNA Technologies (IDT) codon optimization tool. Sequences were ordered as eBlocks (IDT) and cloned into full-length pTT3 derived IgL and IgK expression vectors (Snijder et al., 2018) or subcloned into the pT4-341 HC vector (Mouquet et al., 2010) using InFusion cloning.

Sorts CV3 and PCV1 were directly cloned using Gibson Assembly. Second round PCR primers were adapted to include homology regions that corresponding to the leader sequence and constant regions on the expression vector. Cycling parameters and post-PCR clean-up remained the same. The backbone expression plasmid was amplified using primers specific for the leader sequence and constant regions in 25 μL reactions containing 2x Platinum SuperFi II DNA polymerase, 100 nM 5′ and 3′ primers, 10 ng template DNA, and 21.5 μL Nuclease-free water. The reaction was cycled at 98C for 30 s, 30 cycles of 98C for 10 s, 60C for 10 s, and 72C for 3 minutes and 30 s, followed by 72C for 5 minutes. The reaction was treated with 20 units of dpnI and incubated at 37C for 60 minutes. The reaction was purified using a PCR clean-up kit according to manufacturer’s directions or using. The cloning reaction was performed using 100 ng of second round PCR product, 25 ng of backbone, 1 μL 5x InFusion HD Enzyme and nuclease-free water for a total reaction volume of 3 ul and incubated at 50C for 15 minutes.

The cloning reactions were used to transform OneShot DH5 Alpha cells (according to manufacturer’s directions and plated on agar plates containing ampicillin and grown overnight. Colonies were used to seed 5 mL LB broth cultures containing ampicillin. DNA was prepared using QIAprep Spin Miniprep Kit. Equal amounts of heavy and light chain expression plasmids and a 1:3 ratio of PEI was used to transfect 293-6E cells at a density of 1x10ˆ6 cells/mL in Freestyle 293 media. Supernatants were collected 6 days post transfection by centrifugation at 4,000 g followed by filtration through a 0.22 μM filter. Clarified supernatants were then incubated with Protein A agarose beads overnight followed by extensive washing with 1x PBS. Antibodies were eluted using 0.1M Citric Acid into a tube containing 1M Tris then buffer exchange into 1xPBS using an Amicon centrifugal filter. We recently reported an initial characterization of the anti-S antibody responses generated by CV1 (Seydoux et al., 2020).

#### 10X sequencing

PBMCs were thawed in a 37°C water bath with pre-warmed RPMI + 10% FBS. B cells were isolated from all samples using the EasySep Human B cell Isolation Kit. For the B cell receptor sequencing, cells were partitioned into gel-bead-emulsions and a cDNA was generated with each cell carrying a unique 10x identifier using the Chromium Single Cell 5′ Library and Gel Bead Kit and the Chromium Single Cell A Chip Kit. The cDNA was enriched for V(D)J cDNA using the Human B cell Chromium Single Cell V(D)J Enrichment Kit followed by library construction to add the priming sites used by Illumina sequencers. The V(D)J enriched library was sequenced on an Illumina HiSeq or MiSeq. Data were analyzed using the Loupe V(D)J Browser (v. 3.0.0). 15,000 cells were analyzed per donor yielding 5,000-7,000 clonotypes each. Fred Hutch Genomics core performed the sequencing and the Fred Hutch Bioinformatics core performed processing of the raw sequence data.

#### BLI

All BLI experiments were performed on an Octet Red instrument at 30°C with shaking at 500-1000 rpm. All loading steps were 300 s, followed by a 60 s baseline in KB buffer (1X PBS, 0.01% Tween 20, 001% BSA, and 0.005% NaN_3_, pH 7.4), and then a 300 s association phase and a 300 s dissociation phase in KB. For the binding BLI experiments, mAbs were loaded at a concentration of 20 mg/mL in PBS onto Anti-Human IgG Fc capture (AHC) biosensors. After baseline, probes were dipped in either SARS-CoV2 proteins; SARS-CoV-2 RBD, S-2P, S1, S1 NTD orS2; SARS-CoV proteins; SARS-CoV-RBD or S-2P, or human coronavirus spike proteins; HCoV2-OC43, HKU1, NL63 or 229, at a concentration of 2-0.5 mM for the association phase. The binding of mature VRC01 was used as negative control to subtract the baseline binding in all of these experiments.

#### ACE2 competition BLI

To measure competition between mAb and RBD for ACE2 binding, ACE2-Fc was biotinylated with EZ-Link NHS-PEG4-Biotin at a molar ratio of 1:2. Biotinylated protein was purified using a Zeba spin desalting column. ACE2-Fc was then diluted to 20-83.3 mg/mL in PBS and loaded onto streptavidin biosensors. Following the baseline phase, association was recorded by dipping into a 0.5 mM solution of either SARS-CoV-2 RBD or 0.5 mM solution of SARS-CoV-2 RBD plus mAb. The binding of RBD and mAb to uncoated sensors was used as background binding and was subtracted from each sample. The area under the curve (AUC) of competition was compared to the AUC of the RBD-alone condition. Samples that showed reduced binding are considered competition. Some samples appear to show enhanced binding in the presence of ACE2, perhaps because ACE2 binding stabilizes and exposes their binding sites, these antibodies are considered not competitive with ACE2.

#### mAb competition BLI

To measure competition between individual mAbs for binding to SARS-CoV-2 S-2P and RBD, S-2P and RBD were biotinylated using EZ-Link NHS-PEG4 Biotin at a molar ratio of 1:2/ Biotinylated protein was purified using a Zeba spin desalting column. RBD was loaded onto streptavidin biosensors. For these experiments, following the baseline in KB, the probe was dipped in the first mAb for a first association phase, with this mAb at a saturating concentration of 2 mM. This was followed by a second baseline in KB. The probe was then dipped into the secondary mAb, at a concentration of 0.5 mM for a second association phase, followed by the standard dissociation phase. For a background control, one sample was run with the second mAb identical to the first mAb, to show the residual binding capacity, and this was subtracted from all samples.

To calculate the competition percentage, the binding of the secondary antibodies to RBD or S-2P was also assessed. Here, streptavidin probes were loaded with biotinylated S-2P or RBD, probes were then dipped in the secondary antibody at 0.5 mM for the association phase, before dissociation in KB as normal. As a background control, the binding of mature VRC01 to the RBD or S-2P was assessed and subtracted from all samples. To calculate competition percentage, the area under the curve (AUC) of this binding curve was calculated, along with the AUC of the competition curve. Percent competition was calculated as: (AUCcompetition÷AUCRBDbinding)×100. Full competition was considered when less than 15% binding capacity remained.

#### Neutralization assays

HIV-1 derived viral particles were pseudotyped with full-length wild-type SARS CoV-2 S, SARS CoV-2 S or SARS-CoV-2 B.1.351 SΔ242-243 (Crawford et al., 2020; Seydoux et al., 2020; Stamatatos et al., 2021). The B.1.351Δ242-243 SARS-CoV-2 variant was produced as described previously (Stamatatos et al., 2021) with the D80A, D215G, K417N, E484K, N501Y, D614G and A701V mutations. Briefly, plasmids expressing the HIV-1 Gag and pol (pHDM-Hgpm2), HIV-1Rev (pRC-CMV-rev1b), HIV-1 Tat (pHDM-tat1b), the SARS CoV2 spike (pHDM-SARS-CoV-2 Spike) and a luciferase/GFP reporter (pHAGE-CMV-Luc2-IRES-ZsGreen-W) were co-transfected into 293T cells at a 1:1:1:1.6:4.6 ratio using 293 Free transfection reagent according to the manufacturer’s instructions. The culture supernatant was harvested after 72 hours at 32°C, clarified by centrifugation, filtered, and frozen at −80C.

293 cells stably expressing ACE2 (HEK293T-hACE2) were seeded at a density of 4000 cells/well in a 100 μl volume in flat clear bottom, black walled, tissue culture 96-well plates. The next day, mAbs were initially diluted to 10 or 100 μg/ml in 60 μl of cDMEM in 96 well round bottom plates in duplicate, followed by a 3-fold serial dilution. An equal volume of viral supernatant was added to each well and incubated for 60 min at 37C. Meanwhile 50 μl of cDMEM containing 6 μg/ml polybrene was added to each well of 293T-ACE2 cells (2 μg/ml final concentration) and incubated for 30 min. The media was aspirated from 293T-ACE2 cells and 100 μl of the virus-antibody mixture was added. The plates were incubated at 37°C for 72 hours. The supernatant was aspirated, and cells were lysed with 100 μl of Steady-Glo luciferase reagent (Promega), and luminescence was read on a Fluoroskan Ascent Fluorimeter. CV1-30 was used as a positive control and AMMO 1 (Snijder et al., 2018) was used as a negative control. Control wells containing virus, but no antibody (cells + virus) and no virus or antibody (cells only) were also included on each plate.

% neutralization for each well was calculated as the RLU of the average of the cells + virus wells, minus test wells (cells +mAb + virus) and dividing this result difference by the average RLU between virus control (cells+ virus) and average RLU between wells containing cells alone, multiplied by 100. The antibody concentration that neutralized 50% of infectivity (IC_50_), or serum dilution that neutralized 50% infectivity (ID_50_) was interpolated from the neutralization curves determined using the log(-inhibitor) versus response-variable slope (four parameters) fit using automatic outlier detection in GraphPad Prism software.

The neutralizing activities of CV1-1 and CV1-30 mAbs were also determined with a slightly different pseudovirus-based neutralization assay as previously described (Böttcher et al., 2006; Naldini et al., 1996).

#### Monitoring RBD-binding to 293-ACE2 cells by flow cytometry

8 pmol of biotinylated S-2P with strep tag peptide sequence on C terminus were mixed with 10 pmol of mAb and incubated for 10 min at RT in a round-bottom tissue culture 96-well plate. 200,000 HEK293T-hACE2 cells in 50 μL of cDMEM were then added to each well and the mixture of cells + RBD or S-2P + mAb was incubated for 20 min on ice. Samples were washed once with ice-cold FACS buffer (PBS + 2% FBS + 1 mM EDTA), before staining cells with DY-549-labeled strep-tactin (1:100 dilution) or Allphycocyanin-labeled streptavidin (1:200 dilution). Cells were washed once with FACS buffer, fixed with 10% formalin for 15 min on ice in the dark, and resuspended in 200 μL of FACS buffer to be analyzed by flow cytometry using a LSRII. Control wells were included on each plate and either had no mAb, no RBD or no S-2P, or were unstained. The mean fluorescence intensity (MFI) for each sample was determined and each sample was normalized to the MFI of the no mAb control.

#### Fab purification

Antigen binding fragment (Fab) was generated by incubating IgG with LysC at a ratio of 1 μg LysC per 10mg IgG at 37°C for 18hrs. Fab was isolated by incubating cleavage product with Protein A resin for 1hr at RT. Supernatant containing Fab was collected and further purified by SEC.

#### Crystal screening and structure determination

The CV2-75 Fab and SARS-CoV-2 RBD complex was obtained my mixing Fab with a 2-fold molar excess of RBD and incubated for 90 min at RT with nutation followed by SEC. The complex was verified by SDS-PAGE analysis. The complex was concentrated to 19 mg/mL for initial crystal screening by sitting-drop vapor-diffusion in the MCSG Suite using a NT8 drop setter. Initial crystal conditions were optimized using the Additive Screen. Diffracting crystals were obtained in a mother liquor (ML) containing 0.1 M Tris, pH 7.5, 0.1 M Calcium Acetate, 15% (w/v) PEG 3350, and 4mM glutathione. The crystals were cryoprotected by soaking in ML supplemented with 30% (v/v) ethylene glycol. Diffraction data were collected at Advanced Photon Source (APS) SBC 19-ID at a 12.662 keV. The dataset was processed using XDS (Kabsch, 2010) to a resolution of 2.80Å. The structure of the complex was solved by molecular replacement using Phaser (McCoy et al., 2007) with a search model of SARS-CoV-2 RBD (PDBid: 6xe1) (Hurlburt et al., 2020) and the Fab structure (PDB: 4fqq4FQQ) (Mouquet et al., 2012) divided into Fv and Fc portions. Remaining model building was completed using COOT (Emsley and Cowtan, 2004) and refinement was performed in Phenix (Adams et al., 2004). The data collection and refinement statistics are summarized in Table S3. Structural figures were made in Pymol.

#### Negative-stain EM

SARS-2-CoV S-6P protein was incubated with a three-fold molar excess of CV1-1 Fab for 30 minutes at room temperature. The complex was diluted to 0.03 mg/ml in 1X TBS pH 7.4 and negatively stained with Nano-W on 400 mesh copper grids. For data collection, a Thermo Fisher Tecnai Spirit (120 kV) and an FEI Eagle (4k x4k) CCD camera were used to produce 296 raw micrographs. Leginon (Suloway et al., 2005) was used for automated data collection and resulting micrographs were stored in Appion (Lander et al., 2009). Particles were picked with DogPicker (Voss et al., 2009) and processed in RELION 3.0 (Scheres, 2012).

#### Infection of k18-hACE2 mice with SARS-CoV-2

icSARS-CoV-2 virus (Xie et al., 2020) was diluted in PBS to a working concentration of 2 × 10^5^ pfu/mL. Mice were anesthetized with isoflurane and infected intranasally with icSARS-CoV-2 (50 uL, 1 × 10^4^ pfu/ mouse) in a ABSL-3 facility. Mice were monitored daily for weight loss. At the indicated day post infection, mice were euthanized via isoflurane overdose and lung tissue was collected in Omni-Bead ruptor tubes filled with 1% FBS-HBSS or Tri Reagent. Tissue was homogenized in an Omni Bead Ruptor 24 (5.15 ms, 15 s). To perform plaque assays, 10-fold dilutions of viral supernatant in serum free DMEM were overlaid on Vero-hACE2/TMPRSS2 monolayers and adsorbed for 1 hour at 37°C. After adsorption, 0.8% Oxoid Agarose in 2X DMEM supplemented with 10% FBS and 5% sodium bicarbonate was overlaid, and cultures were incubated for 72 hours at 37°C. Plaques were visualized by removing the agarose plug, fixing the cell monolayer for 15-30 min in 4% paraformaldehyde, and staining with a crystal violet solution (20% methanol in ddH_2_O). RNA was extracted from Tri Reagent using a Direct-zol RNA MiniPrep Kit, then converted to cDNA using the High-capacity Reverse Transcriptase cDNA Kit. RNA levels were quantified using the IDT Prime Time Gene Expression Master Mix, and Taqman gene expression Primer/Probe sets. All qPCR was performed in 384- well plates and run on a QuantStudio5 qPCR system. SARS-CoV-2 viral RNA-dependent RNA polymerase levels were measured as previously described (Vanderheiden et al., 2020). The following Taqman Primer/Probe sets (Thermo Fisher Scientific) were used in this study: Gapdh (Mm99999915_g1).

### Quantification and statistical analysis

#### Sequence analysis

Sequences were analyzed using Geneious software (Version 8.1.9). Identification and alignments to VH/VL genes, quantification of mutations and CDRH3 length were done using V Quest (Brochet et al., 2008). Mutations were counted beginning at the 5′ end of the V-gene to the 3′ end of the 428 FW3.

#### Statistical analysis

All graphs were completed using GraphPad Prism. For column analysis of multiple independent groups one-way ANOVA with Tukey’s multiple comparison test or with Dunnett’s multiple comparison test was used. For grouped analysis two-way ANOVA with Tukey’s or Šídák’s multiple comparison test. Correlations were determined using nonparametric spearmen correlation and p values and nonlinear fit R squared values are reported. Specific details of statistical methods can be found in figure legends for all panels. Specific pe values are reported in legends and p value ranges are reported for every figure in the legend. ^∗^p < 0.05, ^∗∗^p < 0.01, ^∗∗∗^p < 0.001, ^∗∗∗∗^p < 0.0001.

## Supplemental information

Document S1. Figures S1–S7 and Tables S1–S3

Document S2. Article plus supplemental information

## References

[bib1] Adams P.D., Gopal K., Grosse-Kunstleve R.W., Hung L.W., Ioerger T.R., McCoy A.J., Moriarty N.W., Pai R.K., Read R.J., Romo T.D. (2004). Recent developments in the PHENIX software for automated crystallographic structure determination. J. Synchrotron Radiat..

[bib2] Barnes C.O., West A.P., Huey-Tubman K.E., Hoffmann M.A.G., Sharaf N.G., Hoffman P.R., Koranda N., Gristick H.B., Gaebler C., Muecksch F. (2020). Structures of Human Antibodies Bound to SARS-CoV-2 Spike Reveal Common Epitopes and Recurrent Features of Antibodies. Cell.

[bib3] Baum A., Ajithdoss D., Copin R., Zhou A., Lanza K., Negron N., Ni M., Wei Y., Mohammadi K., Musser B. (2020). REGN-COV2 antibodies prevent and treat SARS-CoV-2 infection in rhesus macaques and hamsters. Science.

[bib4] Baum A., Fulton B.O., Wloga E., Copin R., Pascal K.E., Russo V., Giordano S., Lanza K., Negron N., Ni M. (2020). Antibody cocktail to SARS-CoV-2 spike protein prevents rapid mutational escape seen with individual antibodies. Science.

[bib5] Böttcher E., Matrosovich T., Beyerle M., Klenk H.D., Garten W., Matrosovich M. (2006). Proteolytic activation of influenza viruses by serine proteases TMPRSS2 and HAT from human airway epithelium. J. Virol..

[bib6] Brochet X., Lefranc M.P., Giudicelli V. (2008). IMGT/V-QUEST: the highly customized and integrated system for IG and TR standardized V-J and V-D-J sequence analysis. Nucleic Acids Res..

[bib7] Brouwer P.J.M., Caniels T.G., van der Straten K., Snitselaar J.L., Aldon Y., Bangaru S., Torres J.L., Okba N.M.A., Claireaux M., Kerster G. (2020). Potent neutralizing antibodies from COVID-19 patients define multiple targets of vulnerability. Science.

[bib8] Cai Y., Zhang J., Xiao T., Peng H., Sterling S.M., Walsh R.M., Rawson S., Rits-Volloch S., Chen B. (2020). Distinct conformational states of SARS-CoV-2 spike protein. Science.

[bib9] Cao Y., Su B., Guo X., Sun W., Deng Y., Bao L., Zhu Q., Zhang X., Zheng Y., Geng C. (2020). Potent neutralizing antibodies against SARS-CoV-2 identified by high-throughput single-cell sequencing of convalescent patients’ B cells. Cell.

[bib10] Cerutti G., Guo Y., Zhou T., Gorman J., Lee M., Rapp M., Reddem E.R., Yu J., Bahna F., Bimela J. (2021). Potent SARS-CoV-2 neutralizing antibodies directed against spike N-terminal domain target a single supersite. Cell Host Microbe.

[bib11] Chi X., Yan R., Zhang J., Zhang G., Zhang Y., Hao M., Zhang Z., Fan P., Dong Y., Yang Y. (2020). A neutralizing human antibody binds to the N-terminal domain of the Spike protein of SARS-CoV-2. Science.

[bib12] Clausen T.M., Sandoval D.R., Spliid C.B., Pihl J., Perrett H.R., Painter C.D., Narayanan A., Majowicz S.A., Kwong E.M., McVicar R.N. (2020). SARS-CoV-2 Infection Depends on Cellular Heparan Sulfate and ACE2. Cell.

[bib13] Crawford K.H.D., Eguia R., Dingens A.S., Loes A.N., Malone K.D., Wolf C.R., Chu H.Y., Tortorici M.A., Veesler D., Murphy M. (2020). Protocol and Reagents for Pseudotyping Lentiviral Particles with SARS-CoV-2 Spike Protein for Neutralization Assays. Viruses.

[bib14] Davies N.G., Barnard R.C., Jarvis C.I., Kucharski A.J., Munday J., Pearson C.A.B., Russell T.W., Tully D.C., Abbott S., Gimma A. (2020). Estimated transmissibility and severity of novel SARS-CoV-2 Variant of Concern 202012/01 in England. medRxiv.

[bib15] Dong E., Du H., Gardner L. (2020). An interactive web-based dashboard to track COVID-19 in real time. Lancet Infect. Dis..

[bib16] Edara V.V., Floyd K., Lai L., Gardner M., Hudson W., Piantadosi A., Waggoner J.J., Babiker A., Ahmed R., Xie X. (2021). Infection and mRNA-1273 vaccine antibodies neutralize SARS-CoV-2 UK variant. medRxiv.

[bib17] Emsley P., Cowtan K. (2004). Coot: model-building tools for molecular graphics. Acta Crystallogr. D Biol. Crystallogr..

[bib18] Faria N.R., Claro I.M., Candido D., Franco L.A.M., Andrade P.S., Coletti T.M., Silva C.A.M., Sales F.C., Manuli E.R., Aguiar R.S. (2021). Genomic characterisation of an emergent SARS-CoV-2 lineage in Manaus: preliminary findings. Virological.org. https://virological.org/t/genomic-characterisation-of-an-emergent-sars-cov-2-lineage-in-manaus-preliminary-findings/586.

[bib19] Finkelstein M.T., Mermelstein A.G., Parker Miller E., Seth P.C., Stancofski E.D., Fera D. (2021). Structural Analysis of Neutralizing Epitopes of the SARS-CoV-2 Spike to Guide Therapy and Vaccine Design Strategies. Viruses.

[bib20] Gaebler C., Wang Z., Lorenzi J.C.C., Muecksch F., Finkin S., Tokuyama M., Cho A., Jankovic M., Schaefer-Babajew D., Oliveira T.Y. (2021). Evolution of antibody immunity to SARS-CoV-2. Nature.

[bib21] Gavor E., Choong Y.K., Er S.Y., Sivaraman H., Sivaraman J. (2020). Structural Basis of SARS-CoV-2 and SARS-CoV Antibody Interactions. Trends Immunol..

[bib22] Hoffmann M., Kleine-Weber H., Schroeder S., Krüger N., Herrler T., Erichsen S., Schiergens T.S., Herrler G., Wu N.H., Nitsche A. (2020). SARS-CoV-2 Cell Entry Depends on ACE2 and TMPRSS2 and Is Blocked by a Clinically Proven Protease Inhibitor. Cell.

[bib23] Hsieh C.L., Goldsmith J.A., Schaub J.M., DiVenere A.M., Kuo H.C., Javanmardi K., Le K.C., Wrapp D., Lee A.G., Liu Y. (2020). Structure-based design of prefusion-stabilized SARS-CoV-2 spikes. Science.

[bib24] Hurlburt N.K., Seydoux E., Wan Y.H., Edara V.V., Stuart A.B., Feng J., Suthar M.S., McGuire A.T., Stamatatos L., Pancera M. (2020). Structural basis for potent neutralization of SARS-CoV-2 and role of antibody affinity maturation. Nat. Commun..

[bib25] Jackson L.A., Anderson E.J., Rouphael N.G., Roberts P.C., Makhene M., Coler R.N., McCullough M.P., Chappell J.D., Denison M.R., Stevens L.J., mRNA-1273 Study Group (2020). An mRNA Vaccine against SARS-CoV-2 - Preliminary Report. N. Engl. J. Med..

[bib26] Ju B., Zhang Q., Ge J., Wang R., Sun J., Ge X., Yu J., Shan S., Zhou B., Song S. (2020). Human neutralizing antibodies elicited by SARS-CoV-2 infection. Nature.

[bib27] Kabsch W. (2010). Xds. Acta Crystallogr. D Biol. Crystallogr..

[bib28] Koenig P.-A., Das H., Liu H., Kümmerer B.M., Gohr F.N., Jenster L.-M., Schiffelers L.D.J., Tesfamariam Y.M., Uchima M., Wuerth J.D. (2021). Structure-guided multivalent nanobodies block SARS-CoV-2 infection and suppress mutational escape. Science.

[bib29] Kreer C., Zehner M., Weber T., Ercanoglu M.S., Gieselmann L., Rohde C., Halwe S., Korenkov M., Schommers P., Vanshylla K. (2020). Longitudinal Isolation of Potent Near-Germline SARS-CoV-2-Neutralizing Antibodies from COVID-19 Patients. Cell.

[bib30] Lander G.C., Stagg S.M., Voss N.R., Cheng A., Fellmann D., Pulokas J., Yoshioka C., Irving C., Mulder A., Lau P.W. (2009). Appion: an integrated, database-driven pipeline to facilitate EM image processing. J. Struct. Biol..

[bib31] Liu L., Wang P., Nair M.S., Yu J., Rapp M., Wang Q., Luo Y., Chan J.F., Sahi V., Figueroa A. (2020). Potent neutralizing antibodies against multiple epitopes on SARS-CoV-2 spike. Nature.

[bib32] Liu Y., Liu J., Xia H., Zhang X., Fontes-Garfias C.R., Swanson K.A., Cai H., Sarkar R., Chen W., Cutler M. (2021). Neutralizing Activity of BNT162b2-Elicited Serum. N. Engl. J. Med..

[bib33] Lv H., Wu N.C., Tsang O.T., Yuan M., Perera R., Leung W.S., So R.T.Y., Chan J.M.C., Yip G.K., Chik T.S.H. (2020). Cross-reactive Antibody Response between SARS-CoV-2 and SARS-CoV Infections. Cell Rep..

[bib34] McCallum M., De Marco A., Lempp F.A., Tortorici M.A., Pinto D., Walls A.C., Beltramello M., Chen A., Liu Z., Zatta F. (2021). N-terminal domain antigenic mapping reveals a site of vulnerability for SARS-CoV-2. Cell.

[bib35] McCoy A.J., Grosse-Kunstleve R.W., Adams P.D., Winn M.D., Storoni L.C., Read R.J. (2007). Phaser crystallographic software. J. Appl. Cryst..

[bib36] Mercado N.B., Zahn R., Wegmann F., Loos C., Chandrashekar A., Yu J., Liu J., Peter L., McMahan K., Tostanoski L.H. (2020). Single-shot Ad26 vaccine protects against SARS-CoV-2 in rhesus macaques. Nature.

[bib37] Mouquet H., Scharf L., Euler Z., Liu Y., Eden C., Scheid J.F., Halper-Stromberg A., Gnanapragasam P.N., Spencer D.I., Seaman M.S. (2012). Complex-type N-glycan recognition by potent broadly neutralizing HIV antibodies. Proc. Natl. Acad. Sci. USA.

[bib82] Mouquet H., Scheid J.F., Zoller M.J., Krogsgaard M., Ott R.G., Shukair S., Artyomov M.N., Pietzsch J., Connors M., Pereyra F. (2010). Polyreactivity increases the apparent affinity of anti-HIV antibodies by heteroligation. Nature.

[bib38] Naldini L., Blömer U., Gage F.H., Trono D., Verma I.M. (1996). Efficient transfer, integration, and sustained long-term expression of the transgene in adult rat brains injected with a lentiviral vector. Proc. Natl. Acad. Sci. USA.

[bib39] Nielsen S.C.A., Yang F., Jackson K.J.L., Hoh R.A., Röltgen K., Jean G.H., Stevens B.A., Lee J.Y., Rustagi A., Rogers A.J. (2020). Human B Cell Clonal Expansion and Convergent Antibody Responses to SARS-CoV-2. Cell Host Microbe.

[bib40] O’Toole Á., Hill V., Pybus O.G., Watts A., Bogoch I.I., Khan K., Messina J.P., Tegally H., Lessells R.R., Giandhari J., Pillay S., COG-UK Consortium, Network for Genomic Surveillance in South Aftrica, Brazil-UK CADDE Genomic Network, Swiss Viollier Sequencing Consortium, SEARCH Alliance San Diego, National Virus Reference Laboratory, SeqCOVID-Spain, Danish Covid-19 Genome Consortium, Communicable Diseases Genomic Network, Dutch National SARS-Cov-2 Surveillance Program, Division of Emerging Infectious Diseases (2021). Tracking the international spread of SARS-CoV-2 lineages B.1.1.7 and B.1.351/501Y-V2. Virological.org, https://virological.org/t/tracking-the-international-spread-of-sars-cov-2-lineages-b-1-1-7-and-b-1-351-501y-v2/592.

[bib41] Pallesen J., Wang N., Corbett K.S., Wrapp D., Kirchdoerfer R.N., Turner H.L., Cottrell C.A., Becker M.M., Wang L., Shi W. (2017). Immunogenicity and structures of a rationally designed prefusion MERS-CoV spike antigen. Proc. Natl. Acad. Sci. USA.

[bib42] Patel A., Jernigan D.B., 2019-nCoV CDC Response Team (2020). Initial Public Health Response and Interim Clinical Guidance for the 2019 Novel Coronavirus Outbreak - United States, December 31, 2019-February 4, 2020. MMWR Morb. Mortal. Wkly. Rep..

[bib43] Pinto D., Park Y.J., Beltramello M., Walls A.C., Tortorici M.A., Bianchi S., Jaconi S., Culap K., Zatta F., De Marco A. (2020). Cross-neutralization of SARS-CoV-2 by a human monoclonal SARS-CoV antibody. Nature.

[bib44] Rambaut A., Loman N., Pybus O., Barclay W., Barrett J., Carabelli A., Connor T., Peacock T., Robertson D.L., Volz E. (2020). Preliminary genomic characterisation of an emergent SARS-CoV-2 lineage in the UK defined by a novel set of spike mutations. Virological.org. https://virological.org/t/preliminary-genomic-characterisation-of-an-emergent-sars-cov-2-lineage-in-the-uk-defined-by-a-novel-set-of-spike-mutations/563.

[bib45] Robbiani D.F., Gaebler C., Muecksch F., Lorenzi J.C.C., Wang Z., Cho A., Agudelo M., Barnes C.O., Gazumyan A., Finkin S. (2020). Convergent antibody responses to SARS-CoV-2 in convalescent individuals. Nature.

[bib46] Rogers T.F., Zhao F., Huang D., Beutler N., Burns A., He W.T., Limbo O., Smith C., Song G., Woehl J. (2020). Isolation of potent SARS-CoV-2 neutralizing antibodies and protection from disease in a small animal model. Science.

[bib47] Sabino E.C., Buss L.F., Carvalho M.P.S., Prete C.A., Crispim M.A.E., Fraiji N.A., Pereira R.H.M., Parag K.V., da Silva Peixoto P., Kraemer M.U.G. (2021). Resurgence of COVID-19 in Manaus, Brazil, despite high seroprevalence. Lancet.

[bib48] Schäfer A., Muecksch F., Lorenzi J.C.C., Leist S.R., Cipolla M., Bournazos S., Schmidt F., Maison R.M., Gazumyan A., Martinez D.R. (2021). Antibody potency, effector function, and combinations in protection and therapy for SARS-CoV-2 infection in vivo. J. Exp. Med..

[bib49] Scheres S.H. (2012). RELION: implementation of a Bayesian approach to cryo-EM structure determination. J. Struct. Biol..

[bib50] Seydoux E., Homad L.J., MacCamy A.J., Parks K.R., Hurlburt N.K., Jennewein M.F., Akins N.R., Stuart A.B., Wan Y.H., Feng J. (2020). Analysis of a SARS-CoV-2-Infected Individual Reveals Development of Potent Neutralizing Antibodies with Limited Somatic Mutation. Immunity.

[bib51] Shi R., Shan C., Duan X., Chen Z., Liu P., Song J., Song T., Bi X., Han C., Wu L. (2020). A human neutralizing antibody targets the receptor binding site of SARS-CoV-2. Nature.

[bib52] Snijder J., Ortego M.S., Weidle C., Stuart A.B., Gray M.D., McElrath M.J., Pancera M., Veesler D., McGuire A.T. (2018). An Antibody Targeting the Fusion Machinery Neutralizes Dual-Tropic Infection and Defines a Site of Vulnerability on Epstein-Barr Virus. Immunity.

[bib53] Song G., He W.T., Callaghan S., Anzanello F., Huang D., Ricketts J., Torres J.L., Beutler N., Peng L., Vargas S. (2020). Cross-reactive serum and memory B cell responses to spike protein in SARS-CoV-2 and endemic coronavirus infection. bioRxiv.

[bib55] Stamatatos L., Czartoski J., Wan Y.H., Homad L.J., Rubin V., Glantz H., Neradilek M., Seydoux E., Jennewein M.F., MacCamy A.J. (25 Jun 2021). mRNA vaccination boosts cross-variant neutralizing antibodies elicited by SARS-CoV-2 infection. Science.

[bib56] Suloway C., Pulokas J., Fellmann D., Cheng A., Guerra F., Quispe J., Stagg S., Potter C.S., Carragher B. (2005). Automated molecular microscopy: the new Leginon system. J. Struct. Biol..

[bib57] Tai W., Zhang X., He Y., Jiang S., Du L. (2020). Identification of SARS-CoV RBD-targeting monoclonal antibodies with cross-reactive or neutralizing activity against SARS-CoV-2. Antiviral Res..

[bib58] Tegally H., Wilkinson E., Giovanetti M., Iranzadeh A., Fonseca V., Giandhari J., Doolabh D., Pillay S., San E.J., Msomi N. (2020). Emergence and rapid spread of a new severe acute respiratory syndrome-related coronavirus 2 (SARS-CoV-2) lineage with multiple spike mutations in South Africa. medRxiv.

[bib59] Tiller T., Meffre E., Yurasov S., Tsuiji M., Nussenzweig M.C., Wardemann H. (2008). Efficient generation of monoclonal antibodies from single human B cells by single cell RT-PCR and expression vector cloning. J. Immunol. Methods.

[bib60] Tortorici M.A., Beltramello M., Lempp F.A., Pinto D., Dang H.V., Rosen L.E., McCallum M., Bowen J., Minola A., Jaconi S. (2020). Ultrapotent human antibodies protect against SARS-CoV-2 challenge via multiple mechanisms. Science.

[bib61] Vanderheiden A., Ralfs P., Chirkova T., Upadhyay A.A., Zimmerman M.G., Bedoya S., Aoued H., Tharp G.M., Pellegrini K.L., Manfredi C. (2020). Type I and Type III Interferons Restrict SARS-CoV-2 Infection of Human Airway Epithelial Cultures. J. Virol..

[bib62] Volz E., Mishra S., Chand M., Barrett J.C., Johnson R., Geidelberg L., Hinsley W.R., Laydon D.J., Dabrera G., O’Toole Á. (2021). Transmission of SARS-CoV-2 Lineage B.1.1.7 in England: Insights from linking epidemiological and genetic data. medRxiv.

[bib63] Voss N.R., Yoshioka C.K., Radermacher M., Potter C.S., Carragher B. (2009). DoG Picker and TiltPicker: software tools to facilitate particle selection in single particle electron microscopy. J. Struct. Biol..

[bib64] Walls A.C., Park Y.J., Tortorici M.A., Wall A., McGuire A.T., Veesler D. (2020). Structure, Function, and Antigenicity of the SARS-CoV-2 Spike Glycoprotein. Cell.

[bib65] Walsh E.E., Frenck R.W., Falsey A.R., Kitchin N., Absalon J., Gurtman A., Lockhart S., Neuzil K., Mulligan M.J., Bailey R. (2020). Safety and Immunogenicity of Two RNA-Based Covid-19 Vaccine Candidates. N. Engl. J. Med..

[bib66] Wan J., Xing S., Ding L., Wang Y., Gu C., Wu Y., Rong B., Li C., Wang S., Chen K. (2020). Human-IgG-Neutralizing Monoclonal Antibodies Block the SARS-CoV-2 Infection. Cell Rep..

[bib67] Wan Y., Shang J., Graham R., Baric R.S., Li F. (2020). Receptor Recognition by the Novel Coronavirus from Wuhan: an Analysis Based on Decade-Long Structural Studies of SARS Coronavirus. J. Virol..

[bib68] Wang C., Li W., Drabek D., Okba N.M.A., van Haperen R., Osterhaus A.D.M.E., van Kuppeveld F.J.M., Haagmans B.L., Grosveld F., Bosch B.J. (2020). A human monoclonal antibody blocking SARS-CoV-2 infection. Nat. Commun..

[bib69] Wang C., van Haperen R., Gutiérrez-Álvarez J., Li W., Okba N.M.A., Albulescu I., Widjaja I., van Dieren B., Fernandez-Delgado R., Sola I. (2020). Isolation of cross-reactive monoclonal antibodies against divergent human coronaviruses that delineate a conserved and vulnerable site on the spike protein. bioRxiv.

[bib70] Wec A.Z., Wrapp D., Herbert A.S., Maurer D.P., Haslwanter D., Sakharkar M., Jangra R.K., Dieterle M.E., Lilov A., Huang D. (2020). Broad neutralization of SARS-related viruses by human monoclonal antibodies. Science.

[bib71] Weinreich D.M., Sivapalasingam S., Norton T., Ali S., Gao H., Bhore R., Musser B.J., Soo Y., Rofail D., Im J. (2020). REGN-COV2, a Neutralizing Antibody Cocktail, in Outpatients with Covid-19. N. Engl. J. Med..

[bib72] Wibmer C.K., Ayres F., Hermanus T., Madzivhandila M., Kgagudi P., Oosthuysen B., Lambson B.E., de Oliveira T., Vermeulen M., van der Berg K. (2021). SARS-CoV-2 501Y.V2 escapes neutralization by South African COVID-19 donor plasma. Nat. Med..

[bib73] Winkler E.S., Bailey A.L., Kafai N.M., Nair S., McCune B.T., Yu J., Fox J.M., Chen R.E., Earnest J.T., Keeler S.P. (2020). SARS-CoV-2 infection of human ACE2-transgenic mice causes severe lung inflammation and impaired function. Nat. Immunol..

[bib74] Wrapp D., De Vlieger D., Corbett K.S., Torres G.M., Wang N., Van Breedam W., Roose K., van Schie L., Hoffmann M., Pöhlmann S., VIB-CMB COVID-19 Response Team (2020). Structural Basis for Potent Neutralization of Betacoronaviruses by Single-Domain Camelid Antibodies. Cell.

[bib75] Wrapp D., Wang N., Corbett K.S., Goldsmith J.A., Hsieh C.L., Abiona O., Graham B.S., McLellan J.S. (2020). Cryo-EM structure of the 2019-nCoV spike in the prefusion conformation. Science.

[bib76] Wu Y., Wang F., Shen C., Peng W., Li D., Zhao C., Li Z., Li S., Bi Y., Yang Y. (2020). A noncompeting pair of human neutralizing antibodies block COVID-19 virus binding to its receptor ACE2. Science.

[bib77] Wu K., Werner A.P., Koch M., Choi A., Narayanan E., Stewart-Jones G.B.E., Colpitts T., Bennett H., Boyoglu-Barnum S., Shi W. (2021). Serum Neutralizing Activity Elicited by mRNA-1273 Vaccine - Preliminary Report. N. Engl. J. Med..

[bib78] Xie X., Muruato A., Lokugamage K.G., Narayanan K., Zhang X., Zou J., Liu J., Schindewolf C., Bopp N.E., Aguilar P.V. (2020). An Infectious cDNA Clone of SARS-CoV-2. Cell Host Microbe.

[bib79] Yan R., Zhang Y., Li Y., Xia L., Guo Y., Zhou Q. (2020). Structural basis for the recognition of SARS-CoV-2 by full-length human ACE2. Science.

[bib80] Yu J., Tostanoski L.H., Peter L., Mercado N.B., McMahan K., Mahrokhian S.H., Nkolola J.P., Liu J., Li Z., Chandrashekar A. (2020). DNA vaccine protection against SARS-CoV-2 in rhesus macaques. Science.

[bib81] Zost S.J., Gilchuk P., Case J.B., Binshtein E., Chen R.E., Nkolola J.P., Schäfer A., Reidy J.X., Trivette A., Nargi R.S. (2020). Potently neutralizing and protective human antibodies against SARS-CoV-2. Nature.

