# Supplementary material for: Isolation and characterization of cross-neutralizing coronavirus antibodies from COVID-19+ subjects
Source: Cell Rep. 2021 Jun 22;36(2):109353. doi: 10.1016/j.celrep.2021.109353 (PMC8216847; doi:10.1016/j.celrep.2021.109353)
Supplement: Document S1. Figures S1–S7 and Tables S1–S3 [file mmc1.pdf]

Cell Reports, Volume *36*

Supplemental information

Isolation and characterization of cross-neutralizing coronavirus antibodies from COVID-19+ subjects

Madeleine F. Jennewein, Anna J. MacCamy, Nicholas R. Akins, Junli Feng, Leah J. Homad, Nicholas K. Hurlburt, Emilie Seydoux, Yu-Hsin Wan, Andrew B. Stuart, Venkata Viswanadh Edara, Katharine Floyd, Abigail Vanderheiden, John R. Mascola, Nicole Doria-Rose, Lingshu Wang, Eun Sung Yang, Helen Y. Chu, Jonathan L. Torres, Gabriel Ozorowski, Andrew B. Ward, Rachael E. Whaley, Kristen W. Cohen, Marie Pancera, M. Juliana McElrath, Janet A. Englund, Andrés Finzi, Mehul S. Suthar, Andrew T. McGuire, and Leonidas Stamatatos

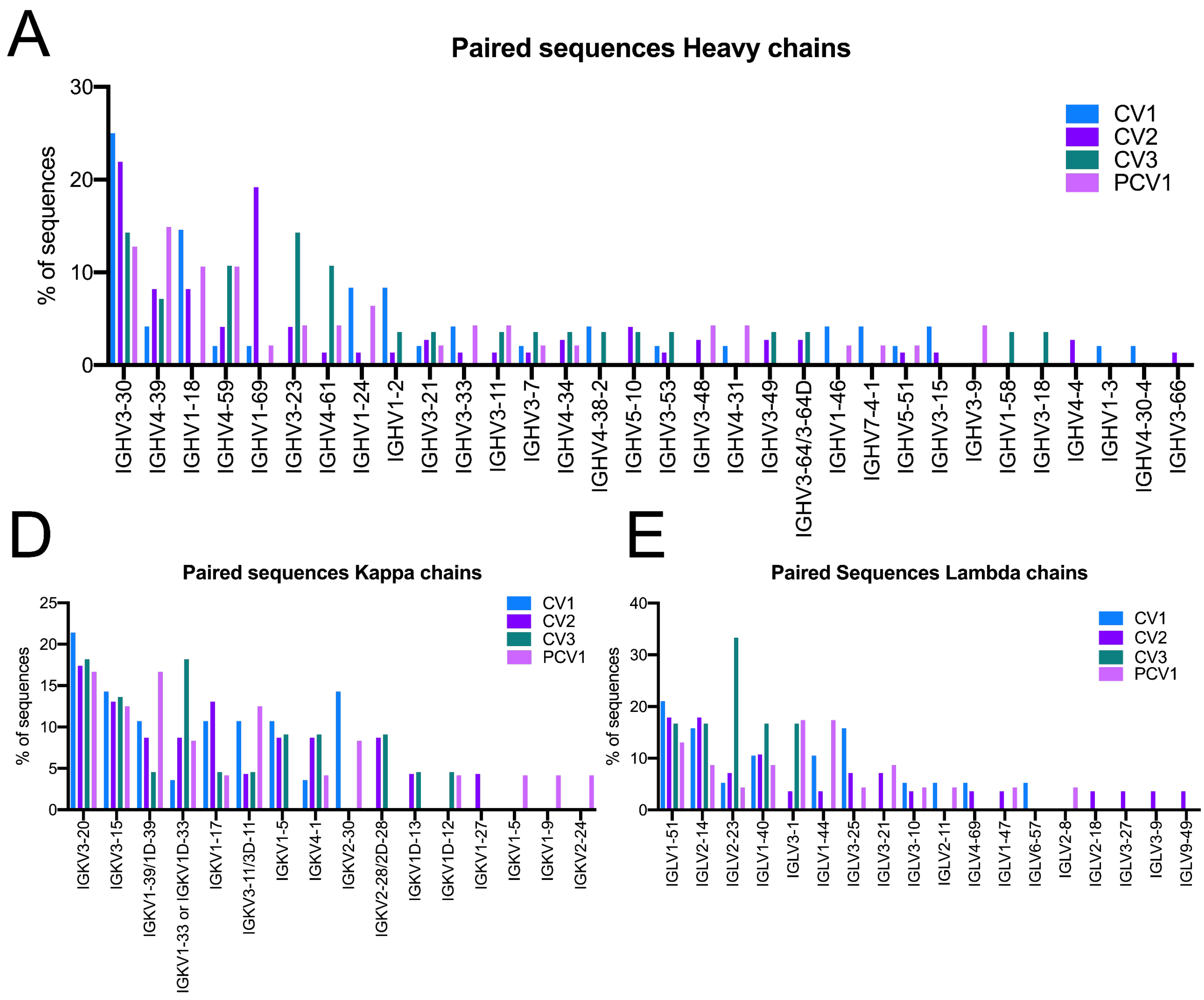

Figure S1: SARS-CoV-2 mAb VH and VL sequencing. Related to Figure 2.

Full sequencing data for all VH and VL genes isolated from the four SARS-CoV-2 positive patients. **(A-C)** Full gene analysis for all paired heavy **(A)**, Kappa **(B)**, and Lambda **(C)** sequences from all four sorts displayed as above as percentage of total sequences from each sort.

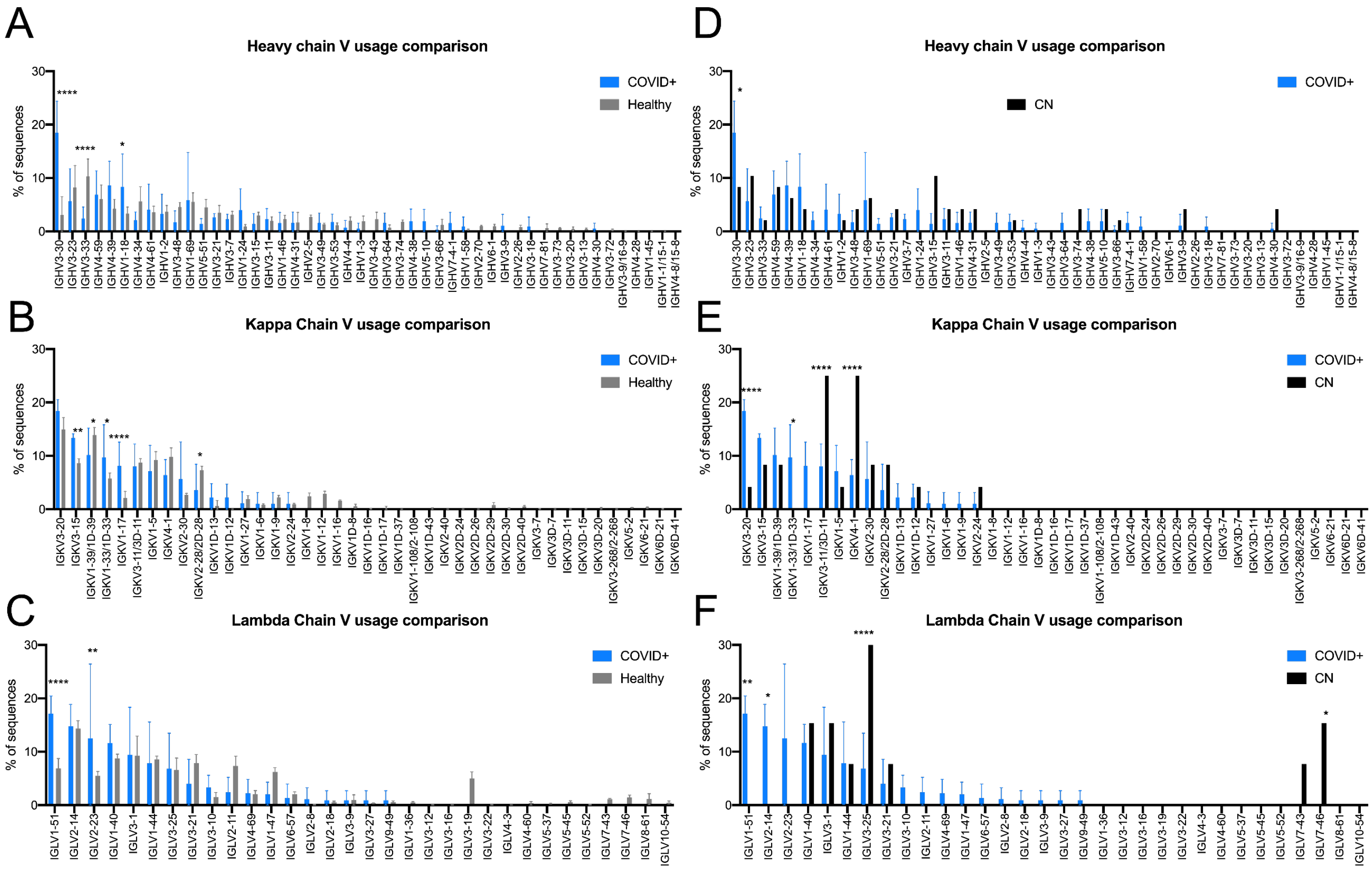

Figure S2: Comparison of VH and VL usage to healthy and naïve repertoire. Related to Figure 2.

The full VH and VL usage in the four SARS-CoV-2 patients was compared to the usage in unbiased sequencing of health individuals and to VH and VL usage by the S-2P+ CN sort. **(A-C)** Comparison of V gene frequencies expressed in total B cells in the 5 10X sorted unexposed individuals to spike specific B cells from COVID+ donors. COVID+ sample V gene frequency is indicated in blue bars while healthy samples are indicated in grey bars for heavy **(A)**, kappa **(B)**, and Lambda **(C)** genes. **(D-F)** Comparison of V gene frequencies from S-2P+ B cells in CN, the naïve individual to spike specific B cells from COVID+ donors. COVID+ sample V gene frequency is indicated in blue bars while naive samples are indicated in black bars for heavy **(D)**, kappa **(E)**, and Lambda **(F)** genes. Significance calculated using two-way-ANOVA. Statistics between different samples evaluated as two-way-ANOVA with Šídák's multiple comparison test. *p<0.05, **p<0.01, ***p<0.001, ****p<0.0001.

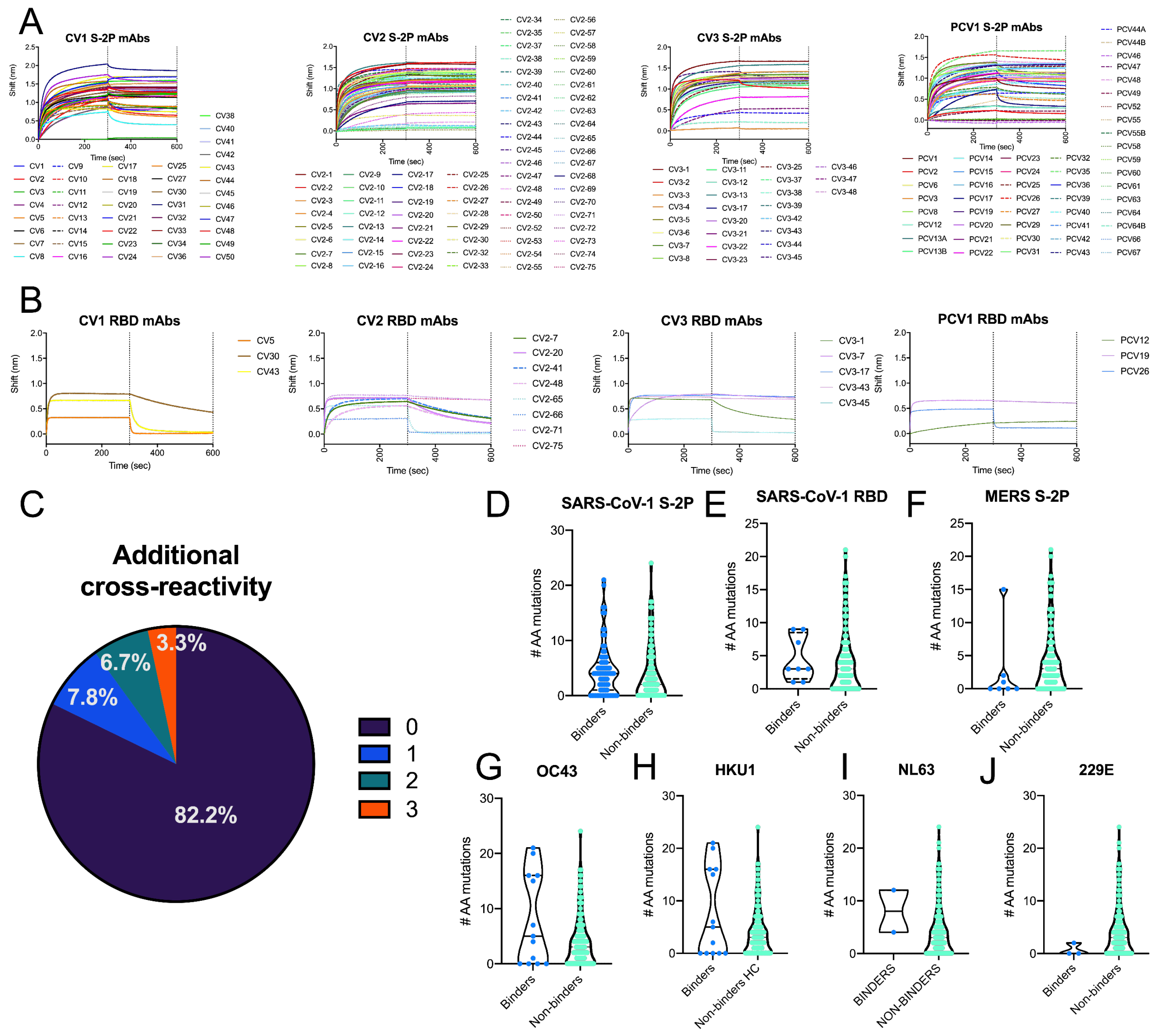

Figure S3: mAb epitope mapping and cross-reactivity BLI. Related to Figure 3.

The binding of the 198 SARS-CoV-2-specific mAbs was assessed by BLI for epitope and cross-reactivity. **(A, B)** All spike-specific mAbs isolated from COVID+ donors were tested by BLI for binding to SARS-CoV-2 S2P **(A)** and SARS-CoV-2 RBD **(B). (C)** Pie chart showing whether mAbs that are cross-reactive against one antigen have additional cross-reactivity with other antigens, either OC43, HKU1, NL63, 229E, MERS or SARS-CoV1. **(D-J)** The number of amino acid mutations (sum of heavy chain and light chain mutations) for each for each SARS-CoV-2 mAb that bound (blue dots) or didn't bind (teal dots) SARS-COV-1 S-2P **(D),** SARS-COV-1 RBD **(E)**, MERS S-2P **(F)**, OC43 **(G)**, HKU1 **(H)**, NL63 **(I)**, or 229E **(J)**. Statistics were assessed by Mann-Whitney test, no comparisons were significant.

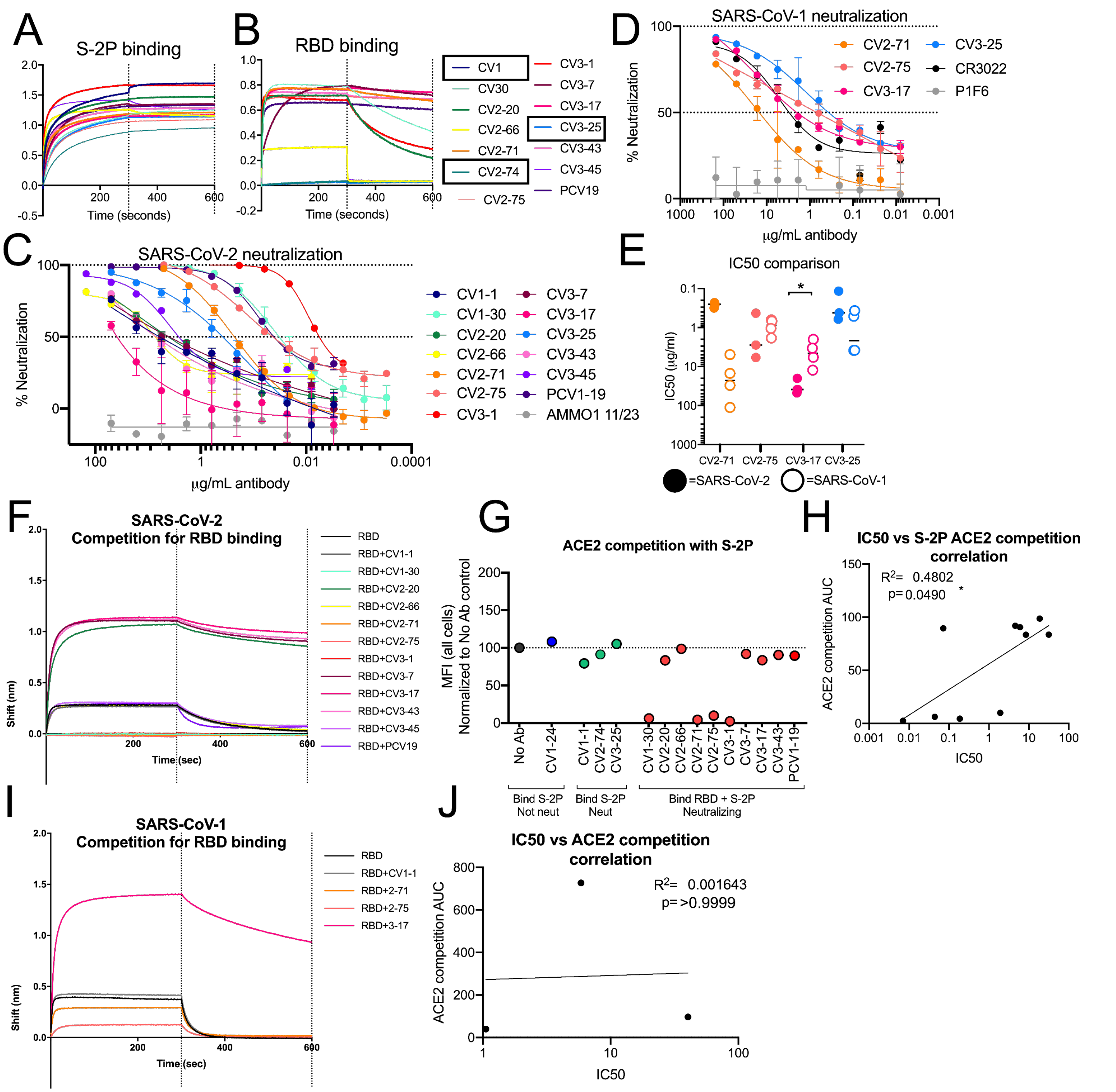

Figure S4: mAb Neutralizing potential and ACE2 competition. Related to Figure 4.

(A) Binding of nAbs to SARS-CoV-2 S-2P as measured by BLI**. (B)** Binding of nAbs SARS-CoV-2 RBD as measured by BLI. Black boxes indicate non-RBD binding nAbs **(C)** Representative neutralization curves for all SARS-CoV-2 nAbs. **(D)** Representative neutralization curves for the indicated mAbs vs SARS-CoV-1. **(E)** Comparison of IC_{50}s for mAbs that bind both SARS-CoV-2 and SARS-CoV-1. Each dot represents an independent replicate. Solid dots represent IC_{50}s for SARS-CoV-2 neutralization while open dots represent IC_{50} for SARS-CoV-1 neutralization. Statistics evaluated by mixed-effect analysis. *$p<0.05$. **(F)** Competition between ACE2 and the indicated mAbs for SARS-CoV-2 RBD binding was measured by BLI. **(G)** Inhibition of fluorescently labeled S-2P binding to ACE2

expressing cells by the indicated nAbs was performed by flow cytometry. The dotted line indicates the MFI of S-2P binding in the absence of mAb. mAbs with values below this line show blocking of ACE2 binding to S-2P. **(H)** Correlation between mAb neutralization IC_{50} and area under the curve (AUC) of competition between mAb and ACE2 for S2P binding. R^2 value for nonlinear fit and Spearmen correlation p value are shown. **(I)** Competition between ACE2 and the indicated mAbs for SARS-CoV-1 RBD binding was measured by BLI. **(J)** Correlation between mAb neutralization IC_{50} and area under the curve (AUC) of competition between mAb and ACE2 for SARS-CoV-1 RBD binding. R^2 value for nonlinear fit and Spearmen correlation p value are shown on graph.

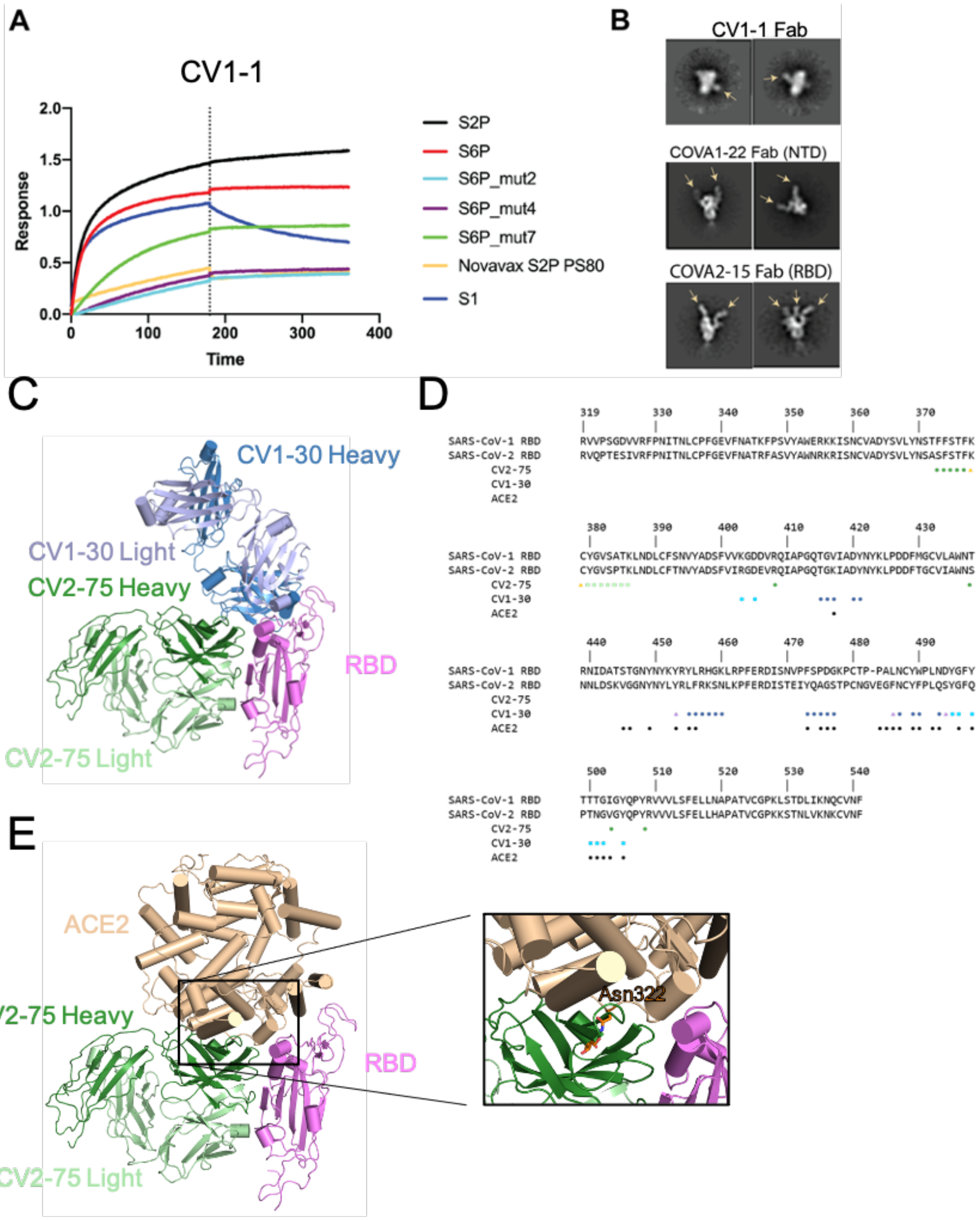

Figure S5: CV1-1 and CV2-75 characterization. Related to Figure 4 and 5.

(A) Biolayer interferometry of immobilized CV1 IgG dipped into recombinant variants of SARS-2-CoV Spike. Vertical dotted line represents transition from association to dissociation steps. 2P = two proline stabilizing mutations, 6P = six proline stabilizing mutations. Mut2, mut4 and mut7 represent additional stabilizing mutations in S1 and/or S2. The Novavax S-2P is formulated with polysorbate 80 and forms nanoparticles

(Bangaru et al., 2020). **(B)** Representative EM 2D class averages from negatively-stained complexes of Fab and recombinant S protein. Arrows indicate Fab densities. COVA1-22 and COVA2-15 represent canonical NTD and RBD targeting antibodies, respectively (Brouwer et al., 2020). **(C,D)** Structural characterization of CV2-75 Fab bound to RBD indicates binding to a cryptic epitope. **(C).** Cartoon representation of CV2-75 Fab (green) bound to RBD (pink) with structure of CV1-30 Fab (blue, PDB ID: 6XE1) superimposed. CV2-75 binds an epitope present only in the "up" RBD conformation. **(D).** SARS-CoV-1 and SARS-CoV-2 RBD sequence alignment indicates that CV2-75 Fab to a conserved region between the two strains. Circles show heavy chain interactions to RBD, squares show light chain interaction, and triangles show both chains. **(E).** RBD-ACE2 (PDB ID: 6M17) superposition to RBD-CV2-75 indicates clashes with glycans at Asn322 of ACE2.

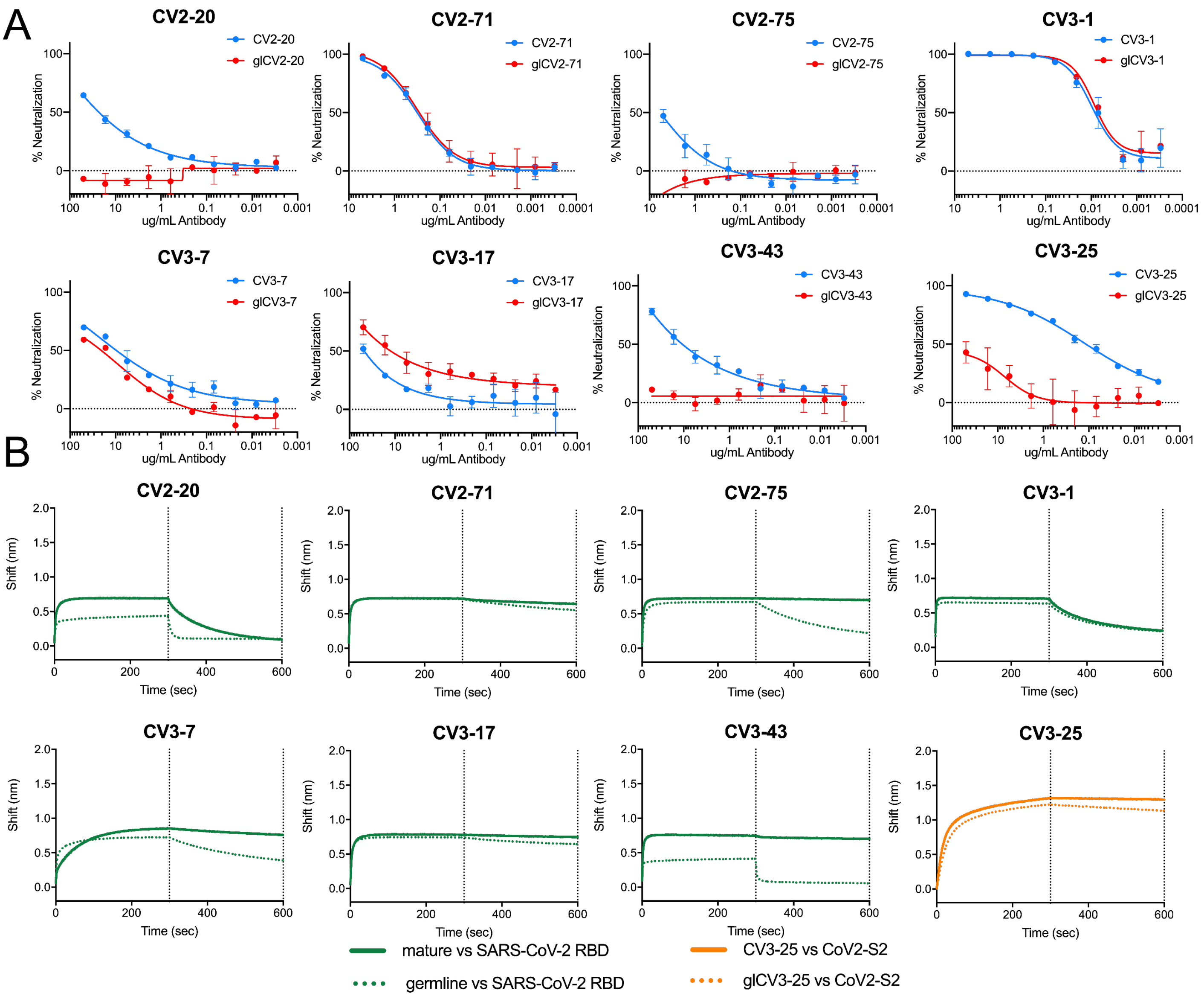

Figure S6: Neutralization potential of Inferred germline versions of mAb. Related to Figure 4.

Versions of Nabs reverted to their germline forms were created and tested for neutralization potential and ability to bind their epitope. **(A)** Neutralization curves for mature (blue) and inferred germline (teal) mAbs. **(B)** The binding of the mature (solid lines) and inferred germline versions of nAbs (dotted lines) to the indicated antigens was measured by BLI. Binding to SARS-CoV-2 S2P (blue) was compared. For CV3-25, binding to SARS-Cov-1 S2 (orange) is shown.

Table S1: B cell sorts from COVID-19 patients. Related to Figure 1 and 2.

Donor	Weeks post symptom onset	PBMCs sorted	B cells sorted	%S-2P++ out of total B cells	%RBD++ out of total B cells	%RBD+ out of S-2P++ B cells	Unpaired Heavy chains	Unpaired Kappa chains	Unpaired Lambda chains	Paired Heavy chains	Paired lambda chains	Paired kappa chains	mAbs produced
**Patient 1 (CV1)**	3	~18 million	768	0.65	0.0352	5	103	97	90	48	20	28	48
**Patient 2 (CV2)**	3.5	~10 million	384	0.37	0.0482	12.7	132	125	112	73	28	45	73
**Patient 3 (CV3)**	6	~10 million	432	0.23	0.0265	13	38	98	70	27	21	6	27
**Patient 4 (PCV1)**	7	~10 million	1736	1.84	0.174	9.4	68	33	31	47	23	24	47
**Healthy sort (CN)[a]**	N/A	~5.7/~5.8 million	70/96	0.104/0.128	0.015/0.019	11.3/9	24/42	37/24	15/15	9/27	2/11	7/16	9/27

a. The healthy sort, CN represents two distinct sorts of the same healthy individual. Values for each individual sort are separated by a forward slash in the table

Table S2: SARS-CoV-2 neutralizing mAbs. Related to Figure 3 and 4. This table shows the 14 neutralizing mAbs isolated and their binding epitopes, neutralization IC50, the VH and VL genes they are derived from, the number of mutations in these genes, the length of their CDRH3 and CDRL3, and whether they bind to SARS-CoV-2, SARS-CoV-1 and the other endemic human coronaviruses.

mAb	Epitope	SARS CoV-2 IC$_{50}$	SARS CoV-1 IC$_{50}$	VH Gene	VL Gene	#AA muts in VH	#AA muts in VL	CDRH3 Length	CDRL3 length	SARS-CoV-2 S-2P	SARS-CoV-2 RBD	SARS-CoV-1 S-2P	SARS-CoV-1 RBD	MERS RBD	OC43	HKU1	NL63	229E
**CV1-1**	S1 NTD	8.2 +/- 4.7	N.A.[b]	IGHV4-38-2	IGLV1-44*01	0	0	17	11	+	-	-	-	-	-	-	-	-
**CV1-30**	RBD	0.044 +/- 0.027	N.A.[b]	IGHV3-53*01	IGKV3-20*01	2	0	12	9	+	+	-	-	-	-	-	-	-
**CV2-20**	RBD	7.1 +/- 9.2	N.A.[b]	IGHV1-2*02	IGLV3-21*02	1	2	19	13	+	+	-	+	-	-	-	-	-
**CV2-66**	RBD	17 +/-17	N.A.[b]	IGHV3-49*03	IGKV3-15*01	0	0	18	8	-	-	-	-	-	-	-	-	-
**CV2-71**	RBD	0.19 +/- 0.10	40 +/- 49	IGHV3-23*01	IGLV1-40*01	3	0	26	11	+	+	+	+	-	-	-	-	-
**CV2-74**	Non-S1/S2	N/A[a]	N.A.[b]	IGHV5-10-1*01	IGKV1-33*01	1	1	14	9	+	-	-	-	-	-	-	-	-
**CV2-75**	RBD	1.7 +/- 2.3	1.1 +/- 0.56	IGHV4-59*01	IGLV3-21*02	2	1	15	11	+	+	+	+	-	-	-	-	-
**CV3-1**	RBD	0.0070 +/- 0.0021	N.A.[b]	IGHV1-58*01	IGKV3-20*01	2	0	16	9	+	+	-	-	-	-	-	-	-
**CV3-7**	RBD	4.6 +/- 3.4	N.A.[b]	IGHV3-30-5*01	IGKV1D-13*01	6	3	17	9	+	+	-	+	-	-	-	-	-
**CV3-17**	RBD	37 +/-11	5.9 +/- 4.6	IGHV3-18	IGKV1-33*01	5	2	20	9	+	+	+	+	-	-	-	-	-
**CV3-25**	S2	0.34 +/- 0.20	2.1 +/- 2.0	IGHV5-51*03	IGKV1D-12*01	5	0	18	9	+	-	+	-	-	+	+	-	-
**CV3-43**	RBD	8.0 +/- 2.4	N.A.[b]	IGHV4-39*01	IGKV1-33*01	7	2	18	5	+	+	-	+	-	-	-	-	-
**CV3-45**	RBD	15 +/-22	N.A.[b]	IGHV1-2*02	IGLV2-14*01	10	7	27	10	+	+	-	-	-	-	-	-	-
**PCV19**	RBD	0.072 +/- 0.043	N.A.[b]	IGHV4-59*03	IGKV1-33*01	3	2	16	9	+	+	-	-	-	-	-	-	-

a. An IC50 was not able to be assigned to CV2-74, as discussed in the text.

b. These mAbs did not neutralize SARS-CoV-1

Table S3. Data collection and refinement statistics for crystal structure. Related to Figure 4 and Figure S5.

	**CV2-75 Fab with SARS-CoV-2 RBD**
**Data collection**	
Space group	$P2_12_12_1$
Cell dimensions	
a, *b*, *c* (Å)	86.37, 127.52, 155.67
α, β, γ (°)	90, 90, 90
Resolution (Å)	49.32-2.80 (2.90 – 2.80)
R_{merge}[a]	0.034 (0.612)
<I/σ(I)>	12.10 (1.44)
$CC_{1/2}$	0.999 (0.566)
Completeness	99.48 (98.13)
Redundancy	2.0 (1.99)
**Refinement**	
Resolution (Å)	49.32 – 2.80 (2.90 – 2.80)
No. unique reflections	42798 (4141)
R_{work}[b]/R_{free}[c]	24.52/28.11 (36.32/40.74)
No. atoms	9874
Protein	9830
Water	16
Ligand	28
B-factors (Å^2)	101.12
Protein	101.19
Water	59.90
Ligand	101.33
RMS bond length (Å)	0.003
RMS bond angle (°)	0.65
**Ramachadran Plot Statistics**[d]	
Residues	1284
Most Favored region	92.89
Allowed Region	6.32
Disallowed Region	0.79
Clashscore	5.06
**PDB ID**	7M3I

[a] $R_{merge} = [\sum_h\sum_i|I_h - I_{hi}|/\sum_h\sum_i I_{hi}]$ where I_h is the mean of I_{hi} observations of reflection *h*. Numbers in parenthesis represent highest resolution shell. [b] R_{factor} and [c] $R_{free} = \sum||F_{obs}| - |F_{calc}|| / \sum|F_{obs}|$ x 100 for 95% of recorded data (R_{factor}) or 5% data (R_{free}). [d] MolProbity reference(Williams et al., 2018)
